# DNA methylation-dependent epigenetic regulation of *Verticillium dahliae* virulence in plants

**DOI:** 10.1007/s42994-023-00117-5

**Published:** 2023-09-20

**Authors:** Yun-Ya Chen, Chen Zhu, Jian-Hua Zhao, Ting Liu, Feng Gao, Ying-Chao Zhang, Cheng-Guo Duan

**Affiliations:** 1 Shanghai Center for Plant Stress Biology and Center of Excellence in Molecular Plant Sciences, Chinese Academy of Science, Shanghai, 200032 China; 2 University of Chinese Academy of Sciences, Beijing, 100049 China; 3 College of Life Sciences, Anhui Normal University, Wuhu, 241000 China; 4 State Key Laboratory of Plant Genomics, Institute of Microbiology, Chinese Academy of Sciences, Beijing, 100101 China; 5 Qilu Zhongke Academy of Modern Microbiology Technology, Jinan, 250000 China

**Keywords:** DNA methylation, *Verticillium dahliae*, Pathogenicity, H3K9me3

## Abstract

As a conserved epigenetic mark, DNA cytosine methylation, at the 5’ position (5-mC), plays important roles in multiple biological processes, including plant immunity. However, the involvement of DNA methylation in the determinants of virulence of phytopathogenic fungi remains elusive. In this study, we profiled the DNA methylation patterns of the phytopathogenic fungus *Verticillium dahliae*, one of the major causal pathogens of *Verticillium* wilt disease that causes great losses in many crops, and explored its contribution in fungal pathogenicity. We reveal that DNA methylation modification is present in *V. dahliae* and is required for its full virulence in host plants. The major enzymes responsible for the establishment of DNA methylation in *V. dahliae* were identified. We provided evidence that DNA methyltransferase-mediated establishment of DNA methylation pattern positively regulates fungal virulence, mainly through repressing a conserved protein kinase VdRim15-mediated Ca^2+^ signaling and ROS production, which is essential for the penetration activity of *V. dahliae*. In addition, we further demonstrated that histone H3 lysine 9 trimethylation (H3K9me3), another heterochromatin marker that is closely associated with 5-mC in eukaryotes, also participates in the regulation of *V. dahliae* pathogenicity, through a similar mechanism. More importantly, DNA methyltransferase genes *VdRid*, *VdDnmt5,* as well as H3K9me3 methyltransferase genes, were greatly induced during the early infection phase, implying that a dynamic regulation of 5-mC and H3K9me3 homeostasis is required for an efficient infection. Collectively, our findings uncover an epigenetic mechanism in the regulation of phytopathogenic fungal virulence.

**Supplementary Information:**

The online version contains supplementary material available at 10.1007/s42994-023-00117-5.

## Introduction

As a conserved mark of epigenetic modifications in eukaryotic cells, DNA methylation plays essential role in genome stability and gene expression. DNA methylation refers to the addition of a methyl group to the 5’ position of cytosine, to form 5-methylctyosine (5-mC), a process catalyzed by specific DNA methyltransferases (DMTs) with *S*-adenosyl-l-methionine (SAM) as the methyl donor. In mammals, 5-mC occurs predominantly on the CG context (Li and Zhang 2014). In plants, cytosine methylation occurs in the context of symmetric CG, CHG (where H represents A, C or T) as well as asymmetric CHH. The establishment and maintenance of DNA methylation in plants is achieved through different mechanisms, depending on the distinct cytosine contexts. Compared to symmetric CG and CHG methylation, which can be catalyzed and maintained through distinct methyltransferases during DNA replication and cell division, asymmetric CHH methylation is de novo established through the 24-nt small interfering RNA (siRNA)-mediated RNA-directed DNA methylation (RdDM) pathway, a plant-specific DNA methylation mechanism (Zhang et al. 2018). However, DNA methylation is not static during the cell cycle, as it can be reversible and dynamic. A specific DNA methylation state is also determined by DNA demethylation, including passive DNA demethylation, which is caused by a lack of DNA methyltransferase or methyl donor during DNA replication, and active DNA demethylation, which is achieved by DNA demethylase-mediated excision of methylated cytosine and the following base excision repair pathway (Zhang et al. 2018). In contrast to plant DNA demethylases, which can recognize and directly remove the 5-mC base, mammal DNA demethylases oxidize 5-mC first and then catalyze base removal (Wu and Zhang 2017; Zhang et al. 2018).

Numerous reports have shown that DNA methylation is involved in many important biological processes, both in mammals and plants, ranging from basal development to environmental responses (Chang et al. 2020; Deleris et al. 2016; Li and Zhang 2014; Xie and Duan 2023; Xie et al. 2023). Despite the extensively explored functions in plants and mammals, fungi also provide an excellent model for understanding the structure and function of chromatin. However, due to the lack of 5-mC modification in yeast, the filamentous fungus *Neurospora crassa*, as a model fungus, provides a rich source for the study of fungal DNA methylation. In *N. crassa*, about 1.5% of cytosine is methylated, and its methylation type is not limited to the symmetric form of CG and CHG, but also asymmetric CHH sites (Goll and Bestor 2005). There are two DNA methyltransferases in *N. crassa*, one of which is RID (RIP defective) that functions in the RIP (Repeat-induced point mutation) process. However, the *rid* mutant shows no significant changes in DNA methylation. In contrast, another DNA methyltransferase, Dim2 (Defective in methylation 2), is not required by the RIP pathway, but is necessary for all known DNA methylation (Kouzminova and Selker 2001). In *N. crassa*, Dim2-mediated DNA methylation is closely associated with histone modification H3K9me3. The H3K9me3 reader protein Hp1 (Heterochromatin protein 1) can interact with Dim2 to promote the establishment of DNA methylation (Honda and Selker 2008; Tamaru and Selker 2001, 2003). In addition, the five core components (Dim5/7/9, CUL4/DDB1) in the DCDC complex (DIM-5/-7/-9, CUL4/DDB1 complex) are also essential for H3K9me3 and DNA methylation (Lewis et al. 2010a, b).

During the last decade, significant attention has been paid to the important participation of DNA methylation in plant immunity (Deleris et al. 2016). Unlike the model fungi, the roles of DNA methylation and associated epigenetic silencing mechanism in pathogenic fungi remain largely elusive. Recently, several studies revealed that DNA methylation is substantially involved in the regulation of fungal development and morphogenetic change (Jeon et al. 2015; So et al. 2018). For example, work on the rice blast disease fungus *Magnaporthe oryzae* indicated that DNA methylation pattern undergoes global reprogramming, during fungal development, and is associated with epigenetic silencing of genes and transposable elements (TEs), and the knockout of a DNA methyltransferase MoDim2 (Deficiency in DNA methylation 2) has substantial impact on *M. oryzae* normal development, but does not affect *M.oryzae* virulence (Jeon et al. 2015). In contrast, although DNA methylation level in *Aspergillus flavus* is negligible in one group’s study (Wang et al. 2012), evidence from another group indicated that the knockout of DmtA methyltransferase made *A. flavus* develop more rapidly on crop seeds compared to the wild strain (Yang et al. 2016), suggesting a role of DNA methylation in *A. flavus* pathogenicity. Despite these limited clues, substantial evidence is still lacking in illustrating the involvement of DNA methylation as a determinant of fungal virulence and the underlying molecular mechanism.

*Verticillium dahliae* is a causal agent of Verticillium wilt diseases, which cause numerous losses in the yield and quality of many economically important crop plants, such as cotton or tomato. In this study, we investigated the DNA methylation of a virulent *V. dahliae* strain, V592, and characterized the function of different DNA methyltransferases. Our data demonstrated that DNA methylation is indispensable for the full virulence of *V. dahliae* in plants. We provided evidence that DNA methyltransferase-mediated establishment of DNA methylation positively regulates fungal virulence, primarily through repressing a conserved protein kinase VdRim15-mediated Ca^2+^ signaling and ROS production, which is essential for the penetration activity of *V. dahliae*.

## Results

### Genome-wide profiling of DNA methylation in virulent *V. dahliae* strain V592

**Fig. 1:**
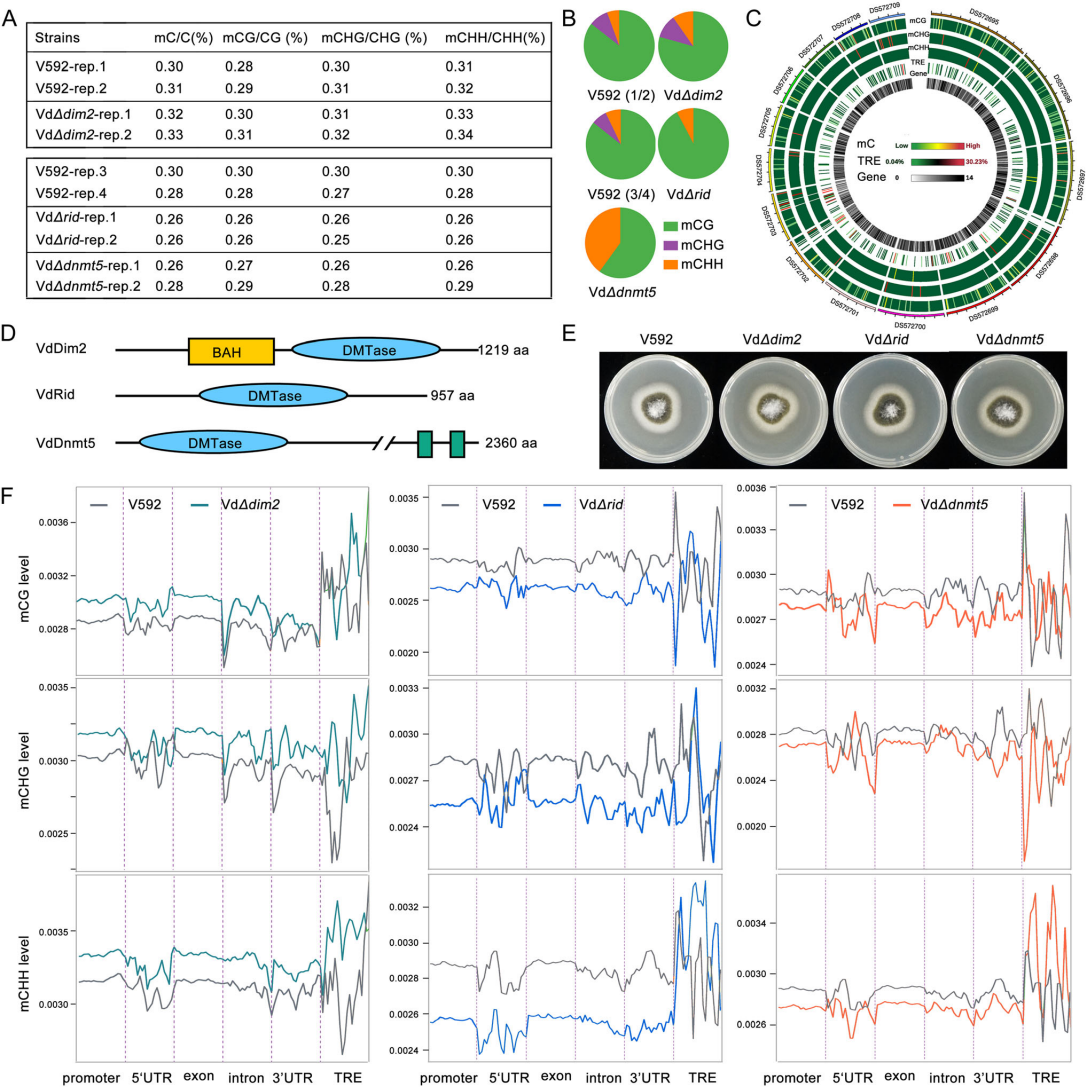
VdRid and VdDnmt5 are the major DNA methyltransferase of *V. dahliae*^V592^. **A** Overall DNA methylation levels in the wild-type V592 and knockout strains at different cytosine contexts. The WGBS assays of Vd*Δdim2* were performed with the same batch as V592 replicate 1 and 2, and the WGBS assays of Vd*Δrid* and Vd*Δdnmt5* were performed with the same batch as V592 replicate 3 and 4. **B** Pie diagrams showing the percentage of DNA methylation at CG, CHG and CHH contexts. **C** Circos diagram showing the methylation density of different chromosomes. **D** The predicted protein domains encoded by the three DNA methyltransferases VdDim2, VdRid and VdDnmt5. Green boxes indicate the Helicase superfamily ATP-binding domain. **E** The morphological phenotype of wild-type V592, Vd*Δdim2,* Vd*Δrid* and Vd*Δdnmt5* mutant strains. Photographs were taken after 14-day-culture. **F** CG (upper panel), CHG (middle panel) and CHH (lower panel) methylation levels at different genomic regions in V592, Vd*Δdim2,* Vd*Δrid* and Vd*Δdnmt5* mutant strains

To confirm the presence of 5-mC in the phytopathogenic fungus *V. dahliae*, a dot blot assay was performed using 5-mC mouse monoclonal antibody in the virulent *V. dahliae* strain V592 (hereafter referred to as V592, Fig. S1), which is isolated from diseased cotton plants (*Gossypium hirsutum*). The results indicated that 5-mC DNA methylation is present in the V592 strain (Fig. S1). Next, we investigated the genome-wide profile of DNA methylation, by performing whole-genome bisulfite sequencing (WGBS) assays, with two biological replicates. WGBS yielded about 14 million raw reads and 13.8 million clean reads. Among these clean reads, 74.48% (mapping rate) of reads were successfully aligned to the reference genome. The whole genome average coverage depth is 72.57X. These results demonstrated that DNA can be methylated at all cytosine contexts (Fig. 1A). The overall methylated cytosine (mC/C) level in V592 was around 0.30%, among which mCG/C, mCHG/C and mCHH/C levels were 0.264%, 0.027% and 0.017%, respectively. which is much lower than that in the model plant *Arabidopsis thaliana* (~ 4.6%) (Kakutani et al. 1999) and model fungus *N. crassa* (~ 1.5%) (Aramayo and Selker 2013). Among three cytosine contexts, CG methylation (85.6%) is the predominant form compared with CHG (8.8%) and CHH methylation (5.6%, Fig. 1B). In contrast, the mean methylation levels of mCG, mCHG and mCHH in respective cytosine context display no obvious difference, and were around 0.30% (Fig. 1A). For the region distribution features, DNA methylation is distributed at distinct genomic regions, including protein coding genes and transposable and repetitive elements (TREs), and TRE regions display higher DNA methylation levels (Fig. 1C, S2 and S3). Collectively, these results suggest that DNA cytosine methylation is conserved in the phytopathogenic fungus *V. dahliae* V592 strain.

### The functionality of different DNA methyltransferases in *V. dahliae* V592 strain

According to a previous study, three DNA methyltransferases were encoded in *Verticillium* spp., including VdDim2, VdRid and VdDnmt5 (Kramer et al. 2021). Consistent with this finding, all three methyltransferases were identified in the V592 strain (Fig. 1D). In addition to the DNA methyltransferase (DMTase) domain shared by all three enzymes, VdDIM2 also possess a bromo-adjacent homology (BAH) domain (Fig. 1D), which is known as a module of chromatin binding (Zhang et al. 2020). In addition, VdDnmt5 also possess a Helicase superfamily ATP-binding domain. To identify the major DMTases required for the establishment of DNA methylation pattern in V592, the knockout strains were generated, via the homologous recombination method (Fig. S4) (Zhu et al. 2022). We first examined the effect of VdDim2, VdRid and VdDnmt5 knockouts on fungal growth. As shown in Fig. 1E and S5, compared with the wild-type V592 strain, the Vd*Δdim2,* Vd*Δrid* and Vd*Δdnmt5* mutant strains displayed normal morphology in hyphal growth, spore number and the production of melanin, indicating that knockouts of *V. dahliae* DNA methyltransferases have no significant effect on fungal growth and development. Next, WGBS assays were performed in Vd*Δdim2,* Vd*Δrid* and Vd*Δdnmt5* mutant strains with two biological replicates. DNA methylation analysis demonstrated that the overall 5-mC levels and the 5-mC levels at different genomic regions were slightly but substantially reduced in both Vd*Δrid* and Vd*Δdnmt5* mutant strains compared with the wild-type V592 strain, and the reductions were comparable between different mC contexts (Fig. 1A, F). Unexpectedly, although certain reductions of 5-mC level were observed at some regions in the Vd*Δdim2* strain, most genomic regions exhibited no significant reduction, even being slightly increased (Fig. 1A, F). This finding is different from a recent report that VdDim2 is the main DNA methyltransferase responsible for DNA methylation in the *V. dahliae* strain JR2 (Kramer et al. 2021), which causes severe wilt disease in crops, including tomato. The functional variation of DNA methyltransferases from different *V. dahliae* species suggests that DNA methyltransferases are still undergoing a rapid evolution in *V. dahliae*. In addition, the proportion of methylated CHG on all methylated cytosine contexts were obviously reduced in the Vd*Δrid* and Vd*Δdnmt5* mutant strains, compared with the wild-type V592 strain, and an obvious reduction in the proportion of mCG was also observed in the Vd*Δdnmt5* mutant (Fig. 1B), suggesting that VdRid and VdDnmt5 have certain preference to CHG methylation. In the model fungus *Neurospora crassa*, the catalyzation of DNA methylation is closely related with the establishment of the repressive heterochromatic mark H3K9me3. DIM2 of *N. crassa* interacts with H3K9me3 reader NcHP1, via its N terminal “PXSTL” motif, to ensure the establishment of DNA methylation (Honda and Selker 2008; Tamaru and Selker 2001, 2003). To decipher the molecular mechanism underlying the weak functionality of V592 VdDim2 in DNA methylation, protein alignment was performed between the VdDim2 amino acid sequence both in V592 and JR2, as well as the homologous protein from *N. crassa* (NcDIM2). Although sharing high sequence similarity (Fig. S6), the N-terminal domain of DIM2 in V592 strain is shorter than that in the JR2 strain and *N. crassa* (Fig. S7A). More importantly, we determined that the PXSTL-like motif is naturally deficient in V592 VdDim2 (Fig. S7A). In line with this finding, our yeast two-hybrid (Y2H) protein interaction result demonstrated that direct interaction was detected between VdHp1 and VdDim2 in JR2, but not in V592 (Fig. S7B). We speculate that the natural variation in the N terminus may be responsible for the weak functionality of VdDim2 in V592. Nonetheless, we cannot rule out the possibility that VdDim2 of V592 is able to catalyze DNA methylation at some specific loci. Therefore, we focused on dissecting the function of VdRid and VdDnmt5 in the following studies.

### DNA methylation positively regulates *V. dahliae* pathogenicity

**Fig. 2:**
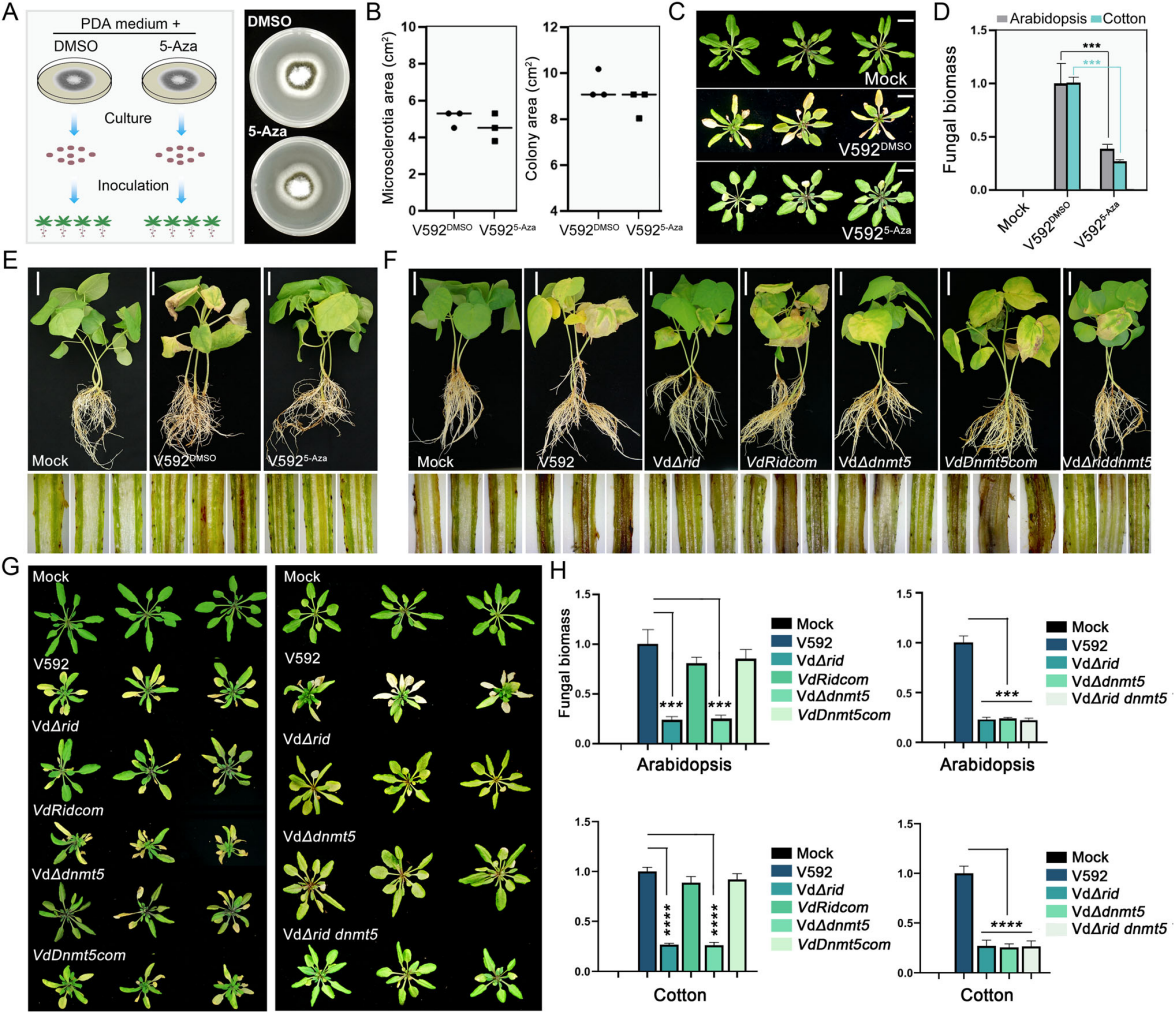
DNA methylation is required for the full virulence of *V. dahliae* strain in plants. **A** Left panel, the diagrammatic sketch (left panel) showing the procedure of 5-Aza treatment on V592 strain and the succedent plant inoculation assay. Right panel, the morphologic phenotype of the 5-Aza and DMSO treated V592 strains on PDA medium. Photos were taken at 14 days after culture. **B** Diagrams showing the microsclerotia (left panel) and colony (right panel) areas of 5-Aza and DMSO treated V592 strains. Data were collected at 14 days after culture. The scatter in the diagram represents three biological replications. **C**, **D** The disease symptoms (**C**) and fungal biomass analysis (**D**) of *A. thaliana* plants (Col-0) inoculated with 5-Aza and DMSO treated V592 strains. Photographs were taken at 21 dpi. Mock represents buffer-inoculated plant controls. An unpaired one-tailed *t* test was performed. ***P < 0.001. Scale bar, 1 cm. **E** The disease symptoms of cotton plants infected with 5-Aza and DMSO treated V592 strains at 21 dpi. The longitudinal section of the stem from inoculated plants were shown. Scale bar, 5 cm. **F**–**H** The disease symptoms and relative fungal biomass analysis (**H**) of cotton (**F**) and *A. thaliana* (**G**) plants infected with the V592, Vd*Δrid*, Vd*Δdnmt5*, *VdRidcom* and *VdDnmt5com* strains at 21 dpi. One-way ANOVA test was performed. ***P < 0.001. ****P < 0.0001

To investigate whether DNA methylation plays a role in the pathogenicity of V592, the DNA methylation inhibitor 5-Aza-2-deoxycytidine (5-Aza) was used to treat V592 on PDA medium (Fig. 2A). The V592 strain treated with dimethyl Sulfoxide (DMSO) served as a parallel control. As shown in Fig. 2A, B, the 5-Aza treatment had no significant effect on the colony growth of *V. dahliae*. After 14 days of culture, both 5-Aza and DMSO treatment strains were subjected to *Arabidopsis thaliana* (Col-0) plant inoculation assay. At 21 days post inoculation, severe disease symptoms were developed in control strain inoculated plants. By contrast, only mild symptoms were developed in *Arabidopsis* plants inoculated with 5-Aza treated V592 (Fig. 2C, D). The reduced virulence of this 5-Aza treatment strain was also observed in the cotton plants (*G. hirsutum*) inoculation assays (Fig. 2E), suggesting that inhibition of DNA methylation results in the attenuation of V592 virulence on plants.

Next, the negative effect of 5-Aza treatment on fungal virulence prompted us to investigate the involvement of DMTases in *V. dahliae* pathogenicity. To this end, *Arabidopsis* and cotton infection assays were performed with the Vd*Δrid* and Vd*Δdnmt5* knockout strains. *VdRid* and *VdDnmt5* complementation strains, under the control of constitutive *Tef* promoter in the background of Vd*Δrid* and Vd*Δdnmt5* mutants, *VdRidcom* and *VdDnmt5com*, were also generated to serve as control strains. The disease symptom observation and fungal biomass analysis results revealed that the virulence of Vd*Δrid* and Vd*Δdnmt5* mutant strains was significantly reduced compared with V592 strain, and complementation of *VdRid* and *VdDnmt5* greatly rescued the attenuated virulence of Vd*Δrid* and Vd*Δdnmt5* mutant strains (Fig. 2F, H). These findings are consistent with the above observation of 5-Aza-dependent inhibition of *V. dahliae* virulence. As a control, Vd*Δdim2* strain was also subjected to inoculation assay. As shown in Fig. S8, the virulence of Vd*Δ*dim2 only displayed weak reduction in comparison with V592. We speculated that VdDim2 may possess some DNA methylation-independent function. Collectively, we concluded that DNA methylation is a positive regulator of V592 virulence in plants.

### DNA methylation negatively regulates the expression of *VdRim15* to promote the full virulence of *V. dahliae* in plants

**Fig. 3:**
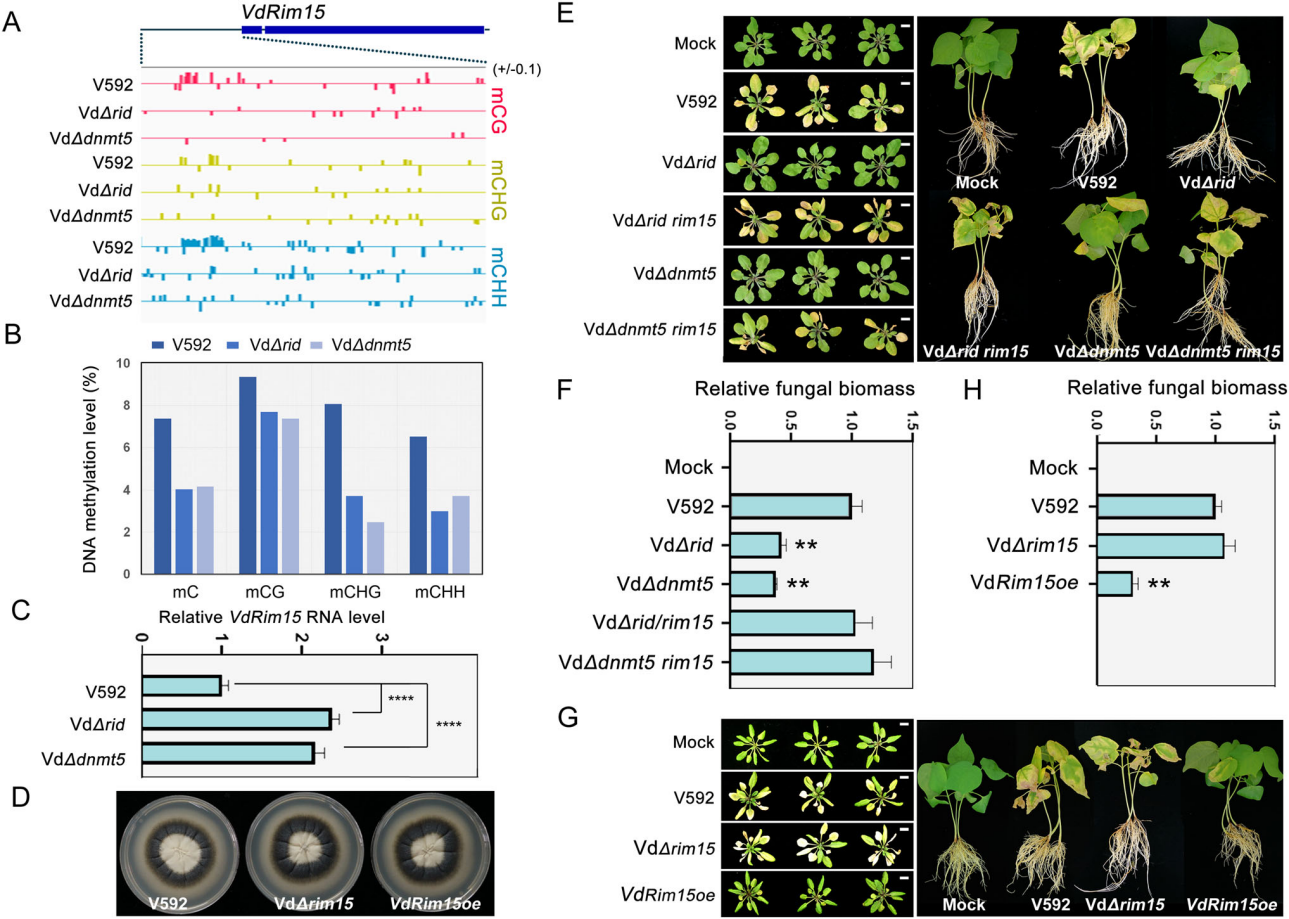
DNA methylation-dependent inhibition of *VdRim15* is required for the full virulence of V592. **A** Snapshot of WGBS showing the DNA methylation levels at *VdRim15* promoter region in V592, Vd*Δrid*, and Vd*Δdnmt5* strains. One representative result of two WGBS replicates was shown for each genotype. **B** The column diagram showing DNA methylation levels at a specific region of Vd*Rim15* promoter in V592, *Δrid* and *Δdnmt5* strains. The DNA methylation levels were calculated based on individual bisulfite sequencing results as shown in Fig. S13. C. RT-qPCR results showing the relative *VdRim15* RNA levels in V592, Vd*Δrid*, and Vd*Δdnmt5* strains. Data are the mean ± SD from three biological replicates. An unpaired one-tailed *t* test was performed. ****p < 0.0001. **D**, **E** The disease symptoms (**E**) and relative fungal biomass analysis (**F**) of *Arabidopsis* and cotton plants inoculated by V592, Vd*Δrid*, Vd*Δdnmt5*, Vd*Δrid rim15*, Vd*Δdnmt5 rim15* strains. Scale bar, 1 cm. F. The morphological phenotype of V592, Vd*Δrim15,* and Vd*Rim15oe* strains. Photographs were taken after 14 days of culture. G-H. The disease symptoms (**G**) and relative fungal biomass analysis (**H**) of *Arabidopsis* and cotton plants inoculated by V592, Vd*Δrim15* and Vd*Rim15oe* strains. The disease symptom photographs were taken at 21 dpi. For fungal biomass analysis, an unpaired one-tailed *t* test was performed. **P < 0.01. Scale bar, 1 cm

Next, to confirm whether DNA methylation has a direct impact on gene expression and uncover the molecular mechanism underlying DNA methylation-mediated regulation of *V. dahliae* virulence, the genes with hypomethylated promoters, regulated by DMTases, were investigated. Due to the much lower methylation level in *V. dahliae*, it was hard to identify differentially methylated regions using classical methods. Even so, we did identify several potential target genes showing reduced promoter 5-mC levels in *Δrid* and *Δdnmt5* mutant strains by searching the WGBS data (Fig. 3A, B and S9A) and by individual bisulfites sequencing confirmation (Fig. 3B). Gene expression analysis, via reverse transcription-quantitative PCR (RT-qPCR), indicated that these genes were significantly up-regulated in Vd*Δrid* and Vd*Δdnmt5* compared with V592 (Fig. 3C and S9B). In line with this result, similar up-regulation of these genes was also observed in 5-Aza treated V592 strains (Fig. S10). These results implied that DNA methylation delivers an epigenetic silencing effect on their expression.

Next, we asked whether there was a correlation between the ectopic expression of these genes and the reduced virulence of Vd*Δrid* and Vd*Δdnmt5*. To answer this question, candidate genes were knocked out in Vd*Δrid* and Vd*Δdnmt5* mutant strains and the generated double knockout strains were subjected to infection assays. Considering that the potential target genes were upregulated in Vd*Δrid* and Vd*Δdnmt5* mutant strains, we assumed that inhibition of target gene expression would be able to rescue the attenuated virulence of Vd*Δrid* and Vd*Δdnmt5* mutant strains. Surprisingly, we observed that knocking out one target gene *VDAG_03223* in Vd*Δrid* and Vd*Δdnmt5* strains could greatly recover the virulence of Vd*Δrid* and Vd*Δdnmt5* mutant strains in *Arabidopsis* and cotton inoculation assays (Fig. 3E, F). *VDAG_03223* encodes a homolog of yeast ScRim15 (hereafter referred to as VdRim15), which is a conserved serine-threonine kinase and functions in multiple processes (Kim 2020; Reinders et al. 1998), including heavy metal stress. In contrast, the reduced virulence of Vd*Δrid* and Vd*Δdnmt5* was not rescued in the double knockout strains of two other target genes (Fig. S11 and S12). To further investigate the role of *VdRim15* in fungal virulence, the Vd*ΔRim15* knockout strain and *VdRim15*-overexpressing strain (Vd*Rim15oe*) were also generated for development observation and inoculation. Both Vd*Δrim15* and Vd*Rim15oe* strains displayed normal colony growth (Fig. 3D). The disease symptom observation and fungal biomass analysis demonstrated that the Vd*Δrim15* mutant strain displayed an almost comparable virulence with V592, whereas the virulence of Vd*Rim15oe* strain was greatly reduced compared with V592 (Fig. 3G, H), a similar phenotype with Vd*Δrid* and Vd*Δdnmt5* strains. Based on these data we proposed that VdRim15 is a negative regulator of *V. dahliae* virulence, and Rid and Dnmt5 positively regulate *V. dahliae* virulence at least partially through conferring DNA methylation-dependent epigenetic silencing of *VdRim15* expression.

### VdRIM15 possesses in vitro kinase activity and is biologically functional in *V. dahliae*

**Fig. 4:**
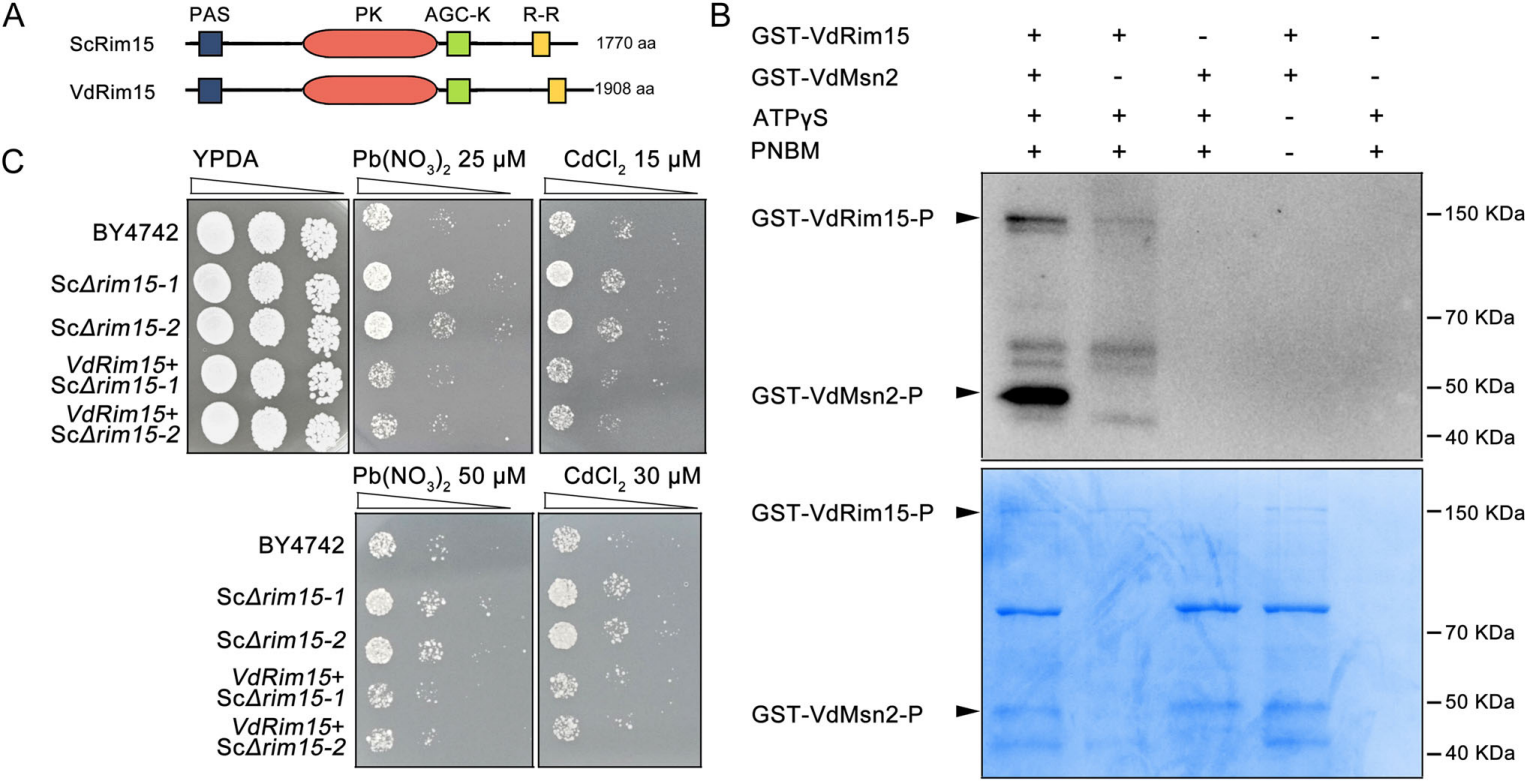
VdRIM15 possesses in vitro kinase activity and is biologically functional in V592. **A** Diagrammatic sketches showing the protein domain structures of Rim in yeast and *V. dahliae.* **B** Autophosphorylation of VdRim15 and phosphorylation of VdMSN2 by recombinant GST–VdRim15. Anti-thiophosphate ester antibody was used to detect phosphorylated proteins. Coomassie staining represents the loading amount of tested proteins. **C** VdRim15 is biologically functional in yeast response to heavy mental stress. Serial dilutions of BY4742, *Δscrim15* mutant strains and VdRim15 over-expression in *Δscrim15* strains were spotted on YPDA plate and SC minimal medium in the presence of 25 and 50 µM Pb(NO_3_)_2_ or 15 and 30 µM CdCl_2_ respectively. Photographs were taken at 4 days post cultivation

VdRim15 belongs to the yeast Greatwall-family protein kinase. In yeast, the PAS kinase ScRim15 was proposed to integrate distinct nutrient-sensing pathway signals and to control transcriptional reprogramming upon nutrient stress (Orzechowski Westholm et al. 2012). VdRim15 has a similar domain composition with its yeast homolog ScRim15 (Fig. 4A), including a N-terminal PAS repeat, a protein kinase domain (PK), an AGC-kinase C-terminal domain (AGC-K) and a Response regulatory domain (R-R). To confirm whether VdRim15 protein kinase domain possesses catalytic activity, an in vitro kinase assay was performed using GST-tagged recombinant protein purified from *Escherichia coli*. In this assay, recombinant VdMsn2 protein, encoded by *VDAG_01718*, the *V. dahliae* homolog of yeast transcription factor ScMsn2 that has been shown as the phosphorylation substrate of ScRim15 kinase (Lee et al. 2013), was also purified as the potential substrate of VdRim15 in kinase assay. As expected, the self-phosphorylation signal of VdRim15 was observed (Fig. 4B), and VdMsn2 could be phosphorylated when VdRim5 was present, suggesting that VdRim15 possesses in vitro protein kinase activity in *V. dahliae*.

Next, we asked whether VdRim15 is biologically functional in V592. It has been reported that disruption of Rim15 in *S. cerevisiae* results in increased tolerance to heavy metal stress (Kim 2020). To confirm the in vivo functionality of VdRim15, ScRim15 knockout yeast strain was generated, and the VdRim15 over-expression strain was constructed in Sc*Δrim15* knockout strain under the control of *GAL1* promoter. Both wild-type BY4742 strain, Sc*Δrim15* and Vd*Rim15*/Sc*Δrim15* were spotted on YPDA medium or SC minimal medium in the presence of 25/50 µM Pb(NO_3_)_2_ or 15/30 µM CdCl_2_, respectively. As shown in the Fig. 4C, compared with the wild-type BY4742 strain which displayed high sensitivity to metal treatment, increased tolerance was observed in the Sc*Δrim15* mutant strains. Importantly, the sensitivity was recovered in the VdRim15 over-expression strains. Based on these results, we propose that VdRim15 is biologically functional in vivo.

### DNA methylation-mediated repression of VdRim15 positively regulates the penetration activity of V592

**Fig. 5:**
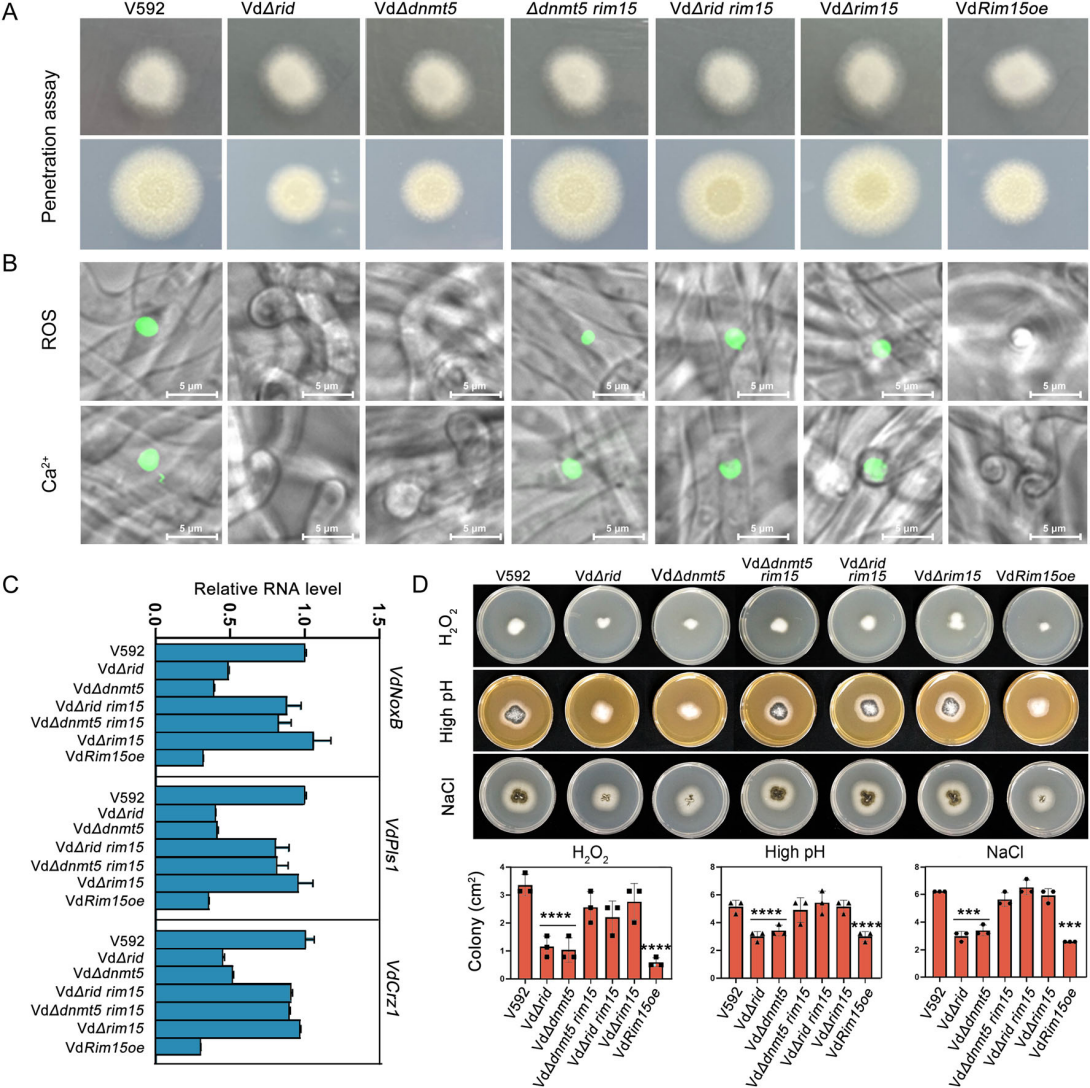
VdRid and VdDnmt5 positively regulate *V. dahliae* penetration activity partially through repression of Vd*Rim15* expression. **A** Penetration ability assay. Colonies of V592, the knockout strains of Vd*Rid*, Vd*Dnmt5*, and Vd*Rim15*, as well as Vd*Rim15oe* strain grown on MM medium overlaid with a cellophane layer (above) and removal of the cellophane membrane (below). Photographs were taken at 3 and 8 dpi, respectively. **B** Detection of ROS production (upper panel) and intracellular Ca^2+^ elevation in different strains. Different strains were grown on MM medium overlaid with the cellophane membrane for 2 days, and ROS production and intracellular Ca^2+^ were detected with DCFH-DA and Fluo-4 AM, respectively. Fluorescence intensity represents the level of ROS and Ca^2+^. Bar = 20 μm. **C** Expression analysis of the VdCrz1 signaling-related genes in V592, Vd*Δrid*, Vd*Δdnmt5*, Vd*Δrid rim15*, Vd*Δdnmt5 rim15*, Vd*Δrim15*, and Vd*Rim15*oe strains by RT-qPCR. Total RNA was isolated from hyphae grown on the cellophane membrane for 2 days. Data are the mean ± SD from three biological replicates. **D** Growth phenotype (upper panel) of different strains under H_2_O_2_ (10 mM), high pH (10) and NaCl (0.5 M) treatments. Photographs were taken at 14 days post treatments. Column diagrams (lower panel) showing the measurement of colony area of different strains under the above stress treatments. For each strain, 3 samples were subjected to measurement of colony area. Each black dot represents a measure event. Gray horizontal lines represent the mean, and the error bars indicate ± SD from the number of measured events (*n* = 3). One-way ANOVA test was performed. ***P < 0.001. ****P < 0.0001

To dissect the roles of DNA methylation-mediated repression of VdRim15 in the full virulence of V592, the penetration ability of hyphae was investigated through a cellophane membrane culture assay as (Zhao et al. 2016). Different strains were incubated on a cellophane membrane laid on minimal medium. As shown in Fig. 5A, fungal hyphae penetration from the cellophane membrane and growth on medium was observed for V592, Vd*Δrim15,* Vd*Δrid rim15* and Vd*Δdnmt5 rim15* strains at 5 dpi. In contrast, the penetration activity was greatly reduced in the knockout mutants of Vd*ΔRid* and Vd*ΔDnmt5*. The colonies of penetrated fungal hyphae on medium were much smaller than V592 strain (Fig. 5A, lower panel). A similar phenotype was also observed for the VdRim15-overexpressing strain (Fig. 5A, lower panel).

Previous study has shown that hyphopodium-specific reactive oxygen species (ROS)-Ca^2+^ signaling is required for the formation of a penetration peg and initial colonization of *V. dahliae* on plant roots (Zhao et al. 2016). In this mechanism, VdNoxB, a catalytic subunit of membrane-bound NADPH oxidase for ROS production, and VdPls1, a tetraspanin, promote ROS production and activate VdCrz1 signaling through Ca^2+^ elevation in hyphopodia to regulate the formation of the penetration peg during the initial colonization on plant roots (Zhao et al. 2016). To test whether the impaired penetration activity observed in Vd*Δrid,* Vd*Δdnmt5* and Vd*Rim15oe* strains was related with mis-regulated Ca^2+^ and ROS accumulation, the accumulation of free calcium and ROS in the cytoplasm of hyphopodia was detected with Fluo-4AM (Zhao et al 2016) and DCFH-DA, respectively. The microscopic imaging results indicated that the V592, Vd*Δrim15,* Vd*Δrid rim15* and Vd*Δdnmt5 rim15* strains obviously displayed Ca^2+^ gradient and ROS accumulation in the hyphopodium (Fig. 5B, lower panel), whereas no Ca^2+^ and ROS accumulation was detected in the hyphopodium of Vd*Δrid,* Vd*Δdnmt5* and Vd*Rim15oe* strains.

To further confirm the correlation between the Ca^2+^ signaling pathway and the penetration defect, we next examined whether the calcineurin-Crz1 signaling pathway, which is activated by Ca^2+^, is involved in VdRid- and VdDnmt5-mediated regulation of penetration peg formation. To this end, the expression of Vd*Crz1*, Vd*NoxB* and Vd*Pls1* in hyphae was examined by RT-qPCR. The results demonstrated that, compared to V592, Vd*Δrid,* Vd*Δdnmt5* and Vd*Rim15oe* strains displayed reduced accumulation of Vd*NoxB*, Vd*Pls1* and Vd*Crz1* RNA, whereas the RNA levels were not significantly changed in Vd*Δrim15,* Vd*Δrid rim15* and Vd*Δdnmt5 rim15* strains (Fig. 5C). Based on these data, we proposed that VdRid- and VdDnmt5-mediated repression of Vd*Rim15* is at least partially required for the formation of the penetration peg through regulating the VdNoxB/VdPls1-dependent ROS-Ca^2+^ signaling in the hyphopodium.

### VdRid and VdDnmt5 knockout strains display increased sensitivity to NaCl, H_2_O_2_ and high pH stresses

In addition to the role in the formation of penetration peg, we also investigated the response of VdRid and VdDnmt5 knockout strains to several stress treatments, including NaCl, H_2_O_2_ and high pH (10). In the treatment of NaCl, H_2_O_2_ and high pH, the Vd*Δrid* and Vd*Δdnmt5* strains displayed significantly reduced colony growth (Fig. 5D), indicating that VdRid and VdDnmt5 have a positive role in regulating *V. dahliae* tolerance against these stresses. Intriguingly, the reduced growth observed in Vd*Δrid* and Vd*Δdnmt5* strains can be greatly rescued by the dysfunction of VdRim15. Moreover, Vd*Rim15* knockout strain had a similar colony growth with V592, whereas the growth of Vd*Rim15oe* was greatly inhibited under stress treatments. These results suggested that VdRid and VdDnmt5 regulate *V. dahliae* tolerance against these stresses partially through the repression of Vd*Rim15* expression.

### Heterochromatic mark H3K9me3 represses the expression of *VdRim15* to promote the full virulence of *V. dahliae* in plants

**Fig. 6:**
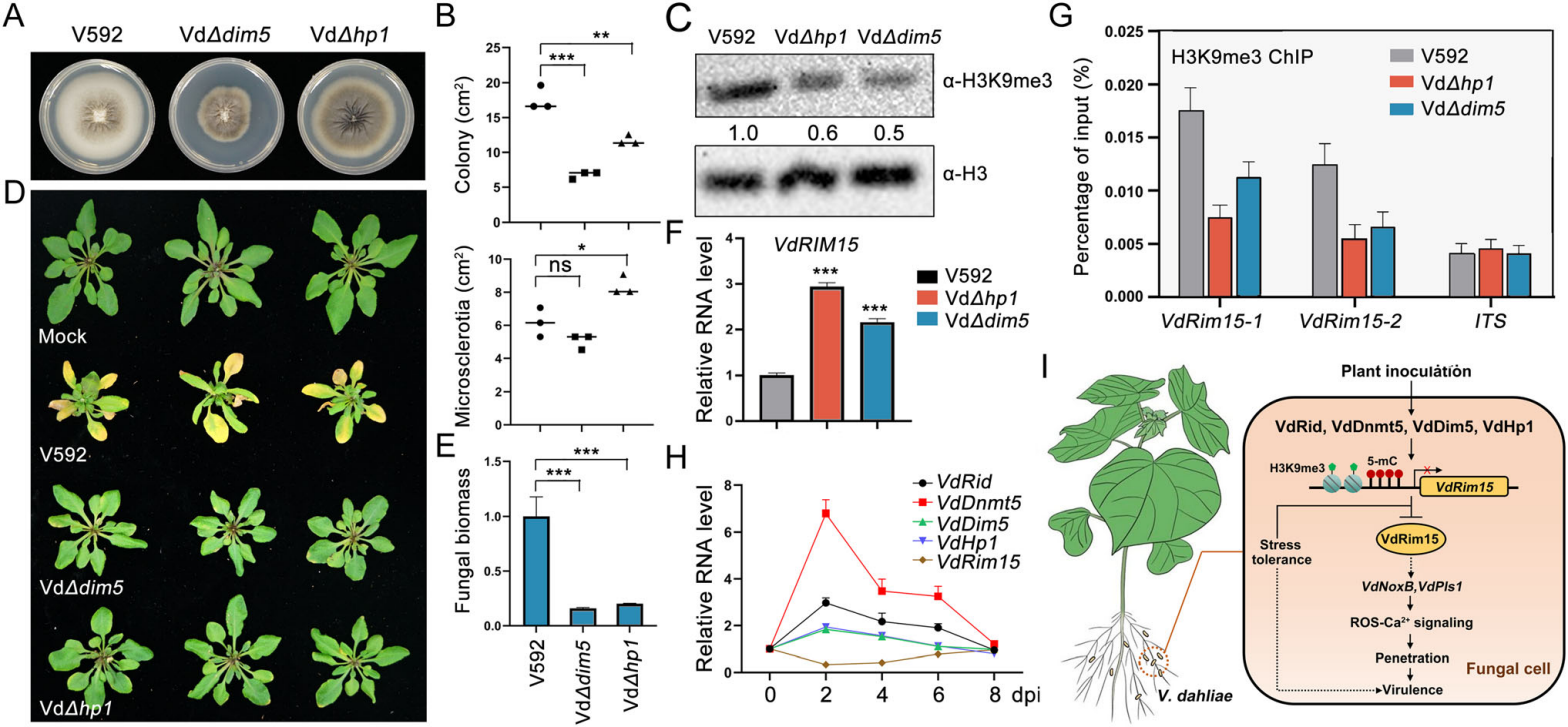
H3K9me3 represses the expression of *VdRim15* to promote the full virulence of *V. dahliae* in plants. **A** Morphological phenotype of V592, *Δdim5* and *Δhp1* knockout mutant strains on PDA plates after 2-week-culture. **B** Quantitation of colony and microsclerotia area in V592, Vd*Δdim5* and Vd*Δhp1* strains. Each black dot represents a measure event from sample, and black horizontal lines represent the mean area. Asterisks indicate significant differences. One-way ANOVA test was used. *P < 0.05; **P < 0.01; ***P < 0.001. *ns* no significance. **C** Immunoblotting result showing the H3K9me3 levels in V592, Vd*Δdim5* and Vd*Δhp1* strains. H3 levels were examined as protein loading control. **D**, **E** Disease symptoms (**D**) and fungal biomass analysis (**E**) of *Arabidopsis* (Col-0 ecotype) inoculated with V592, Vd*Δdim5* and Vd*Δhp1* mutant strains at 21 dpi. Data are mean ± SD from three biological replicates. ***P < 0.001. **F** RT-qPCR result showing the relative RNA levels of *VdRim15* in V592, Vd*Δdim5* and Vd*Δhp1* strains. Data are the mean ± SD from three biological replicates. ***P < 0.001. **G** ChIP-qPCR results showing the relative H3K9me3 levels at two *VdRim15* promoter regions in V592, Vd*Δdim5* and Vd*Δhp1* mutant strains. *ITS* serves as control locus. Data are the mean ± SD from three biological replicates. **H** RT-qPCR results showing the relative RNA levels of selected fungal genes during V592 infection in *Arabidopsis*. Samples were collected at 0, 2, 4, 6, and 8 dpi. **I** A proposed working model of DNA methylation- and H3K9me3-dependent epigenetic regulation of *V. dahliae* virulence

DNA methylation and H3K9me3 are two conserved heterochromatic marks, which are generally associated with transcriptional silencing. As mentioned in the introduction, in *N. crassa*, H3K9me3 is catalyzed by histone methyltransferase NcDim5, and the NcHp1 reads H3K9me3 mark and recruits NcDim2 to catalyze DNA methylation (Honda and Selker 2008). In this study, DNA methylation-mediated repression of *VdRim15* directed us to investigate whether H3K9me3 also plays a role in the repression of *VdRim15* expression and dependent virulence regulation. To this end, the knockout strains of VdDim5 and VdHp1, encoded by genes *VDAG_07826* and *VDAG_05590*, respectively, were generated. Vd*Δdim5* and Vd*Δhp1* strains displayed severe growth and developmental defects (Fig. 6A, B). Immunoblotting results indicated that H3K9me3 levels were greatly reduced in both knockout mutants, suggesting that VdDim5 is functional in V592 (Fig. 6C). Plant inoculation assay indicated that, compared with V592, the Vd*Δdim5* and Vd*Δhp1* knockout strains caused only very weak wilt disease symptoms on *Arabidopsis *(Fig. 6D, E). Intriguingly, similar to the upregulation observed in the knockout strain of Vd*Rid* and Vd*Dnmt5*, Vd*Rim15* RNA level was greatly increased in the Vd*Δdim5* and Vd*Δhp1* knockout strains compared with V592 (Fig. 6F), suggesting that VdDim5 and VdHp1 negatively regulate *VdRim15* expression. Based on these findings, we proposed that the reduced virulence of Vd*Δdim5* and Vd*Δhp1* knockout strains may be partially attributed to the upregulation of *VdRim15* through regulation of H3K9me3 deposition. To test this hypothesis, a H3K9me3 chromatin immunoprecipitation quantitative PCR assay was performed in V592, Vd*Δdim5* and Vd*Δhp1* strains. Two Vd*Rim15* promoter regions were selected to examine H3K9me3 levels. As shown in Fig. 6G, H3K9me3 levels at these two promoter regions were significantly reduced in Vd*Δdim5* and Vd*Δhp1* strains compared with V592, whereas levels were not significantly changed at control *ITS* locus. These results suggest that VdDim5 and VdHp1 promote H3K9me3 deposition at the *VdRim15* promoter and confer its epigenetic silencing.

Epigenetic modifications are generally considered to be dynamically regulated during the life cycle. Therefore, we asked how were these heterochromatin genes regulated during *V. dahliae* infection. To answer this question, V592 inoculation assay was performed in *Arabidopsis* and fungal genes were examined at different time points post inoculation. Intriguingly, the RNA levels of these heterochromatin genes, including *VdRid*, *VdDnmt5*, *VdDim5* and *VdHp1*, were significantly increased at 2 dpi, and slowly returned to original levels over the following days (Fig. 6H). In contrast to these genes, the expression of Vd*Rim15* was significantly reduced at 2 dpi and subsequently slowly recovered (Fig. 6H). Based on these data, we proposed that DNA methylation and H3K9me3-dependen tepigenetic repression of *VdRim15* expression is required for the full virulence of *V. dahliae* during early infection (Fig. 6I).

## Discussion

Early in 1984, DNA methylation patterns were investigated in a number of fungal strains (Antequera et al. 1984). DNA methylation is one of the most deeply studied epigenetic modifications. Previous studies have shown that DNA methylation plays an essential role in many life processes, ranging from microorganisms to plants and mammals. In this study, our data support DNA methylation as a positive regulator of the pathogenicity of the phytofungal *Verticillium dahliae* V592 strain. We confirmed the presence of 5-mC modification in *V. dahliae* V592 and investigated the functionality of three methyltransferases. We show that VdRid and VdDnmt5, and possibly VdDIM2, both contribute to the establishment of DNA methylation and is required for the full virulence of V592 in plants. We identified Vd*Rim15*, a protein kinase gene, as a target of DNA methylation-mediated transcriptional repression, and revealed that DNA methylation positively regulates fungal penetration activity, partially by inhibiting the expression of Vd*RIM15*, thereby influencing the virulence in host plants. Our findings demonstrate the important participation of DNA methylation in fungal pathogenicity and suggest that DNA methylation dynamics is an important regulatory step during fungal infection.

Recently, increasing evidence has shown that DNA methylation contributes to the virulence of pathogenic fungi. As in the human pathogenic fungus, *Cryptococcus neoformans*, the only DNA methyltransferase DNMT5 interacts with HP1 homologous protein Swi6 to recognize H3K9 methylation. DNMT5 is also proven to be essential to maintain the full virulence of *C. neoformans* (Wang et al. 2022). In addition to human pathogenic fungi, limited studies indicate that DNA methylation can also play important roles in phytopathogenic fungi. The number and species of DNA methyltransferases vary greatly among different phytopathogenic fungi, which are mainly divided into two branches from an evolutionary the perspective. The first branch is a DNMT1 branch, which includes DIM2, DNMT1, DNMT5 and RID. The second branch is a DNMT2 branch, mainly including tRNA-Asp methyltransferase (Bewick et al. 2019). It is reported that the DNA methylation level of phytopathogenic fungi is low, from below the detection threshold to just above the detection threshold of CG methylation and non-CG methylation (Ikeda et al. 2013). At present, the molecular mechanism for the establishment and maintenance of DNA methylation in phytopathogenic fungi is still unclear. Many studies have reported that DNA methylation participates in the development of phytopathogenic fungi. In rice blast fungus, DNA methylation not only plays a role in genome defense, but also makes a vital contribution to normal development. During the whole developmental cycle of *Magnaporthe grisea*, the methylome undergoes a global reprogramming. The DNA methyltransferase DIM2 knockout strain exhibits a fluffier colony than the wild type, and the ability to produce spores is also weakened, but the full virulence is not affected (Jeon et al. 2015). However, other studies have shown that treatment of *Aspergillus flavus* with methylation inhibitors will lead to morphological changes. The DNA methyltransferase knockout strain of *A. flavus* develops faster on peanut seeds and corn grains than the wild type, suggesting that DNA methylation may negatively regulate its pathogenicity (Wang et al. 2012). These cases indicate that the regulatory mechanisms of DNA methylation on pathogenicity in different pathogenic fungi are diverse.

Three putative DNA methyltransferases-VdDim2, VdRid and VdDnmt5 have been reported in *Verticillium dahliae* (Kramer et al. 2021)*.* In our work, using whole genome bisulfite sequencing, we identified that knocking out *VdRid* and *VdDnmt5* lead to a substantial reduction of methylation level (Fig. 1). Surprisingly, knocking out *VdDim2* in V592 did not lead to a reduction of 5-mC, but a slight increase, which is different from the previous study that NcDIM2 is the only functional DNA methyltransferase in *Neurospora crassa*. Subsequent experiments find that VdDim2 in *V. dahliae* V592 cannot interact with H3K9me3 reader HP1 due to its lack of PXSTL motif (Fig. S7). We speculate that the selective pressure of the environment may force VdDim2 to undergo such a variation so as to retain its other important functions. In line with this notion, overexpressing JR2-Dim2 in Vd*Δdnmt5* mutant greatly rescued the up-regulation of VdRim15 gene caused by VdDnmt5 dysfunction (Fig. S14), further supporting our conclusion that VdDim2 possess weak function in V592. Intriguingly, both VdRid and VdDnmt5 cannot interact with VdHP1 in V592 strain (Fig. S7C). However, we cannot rule out the possibility that other unknown factors may be able to mediate the association of DNA methyltransferases with H3K9me3 modifiers. In addition, we found that both DNA and H3K9me3 methyltransferases genes were up-regulated in Vd*Δdim2* mutant (Fig. S15), implying that the slight increase of DNA methylation level in Vd*Δdim2* may be due to an indirect effect.

Different from the observation in *M. oryzae* that DNA methylation affects its development rather than pathogenicity, impairment of *V. dahliae* DNA methyltransferases lead to reduced pathogenicity but normal growth. We further revealed that *VdRim15* is a target of VdDnmt5- and VdRid-mediated DNA methylation, which confers a transcriptional repression of *VdRim15*. More importantly, the high expression of *VdRim15* caused by the dysfunctions of VdDnmt5 and VdRid is partially responsible for the antagonized virulence in plants. In yeast, ScRIM15 is reported to possess protein kinase activity, and plays important role in autophagy and nutritional stress (Orzechowski Westholm et al. 2012). Our data also indicate that VdRIM15 possesses protein kinase activity (Fig. 4). The ectopic expression of *VdRim15* in yeast could still respond to heavy metal stress, suggesting that VdRIM15 may have a conserved biological function in *V. dahliae* V592 (Fig. 4). Due to the lack of 5-mC modification in yeast, *Rim15* is supposed to be differently regulated in yeast and *V. dahliae*, which also suggests that *VdRim15* may have other functions in *V. dahliae*. As the first step of infecting plants, colonization has been reported to play a crucial role in the pathogenicity of phytofungi. Hyphopodium-specific ROS-Ca^2+^ signaling has been shown to be essential for the colonization ability of *V. dahliae*. Our data revealed that overexpression of *VdRim15* resulted in a significant reduction of ROS and Ca^2+^ levels in *V. dahliae* and a down-regulation of the expression of penetration peg formation-related genes (Fig. 5). Although these genes may not be the direct substrate of VdRim15-mediated phosphorylation, the possibility cannot be ruled out that other unknown factors involved in ROS-Ca^2+^ signaling may be regulated. In addition to the penetration ability, the response of *V. dahliae* to environmental stress was also influenced by the dysfunction of VdRid and VdDnmt5, and by the high expression of *VdRim15*, which may also have important influence on fungal pathogenicity. Considering Rim15 is a component of the nutritional perception pathway in yeast, whether nutritional perception also plays a role in the formation of virulence during fungal infection is worthy of further exploration. We speculated that the fungus may perceive the nutritional, change during infection to activate/inhibit Rim15, and by the subsequent phosphorylating/dephosphorylating of unknown transcription factor substrates, regulate the expression of the ROS and Ca^2+^ pathways. The specific mechanism needs to be resolved by future studies.

In eukaryotes, DNA methylation is closely associated with histone modification H3K9me3 in constitutive heterochromatin region (Aramayo and Selker 2013). In line with this notion, 5-mC dot blot result demonstrated that DNA methylation levels were obviously reduced in the Vd*Δhp1* and Vd*Δdim5* mutant strains (Fig. S16A), although no direct interaction was observed between VdHP1 and DNA methyltransferases (Fig. S7). By contrast, H3K9me3 levels were not significantly changed in DNA methyltransferase mutants (Fig. S16B). These results suggest that H3K9me3 deposition occurs upstream of DNA methylation. In this work, we showed that H3K9me3 is deposited in the promoter region of *VdRim15*, and acts together with 5-mC to negatively regulate Vd*Rim15* expression, suggesting that histone modifications also play important roles in the formation of *V. dahliae* virulence. Intriguingly, knockout mutants of VdDim5 and VdHp1 displayed severe development defects, indicating that H3K9me3 has other important functions in *V. dahliae*.

A recent study showed that the histone modifications of rice blast fungus undergo a dynamic change during host inoculation (Zhang et al. 2021). In line with this phenomenon, our data indicate that the RNA levels of DNA methyltransferase and H3K9me3-related genes, including Vd*Rid*, Vd*Dnmt5*, Vd*Dim5* and Vd*Hp1*, also underwent dynamic changes during interaction with host plants. These genes display a remarkable upregulation during the early infection process but become slowly reduced afterwards. Intriguingly, the expression of *VdRim15* exhibits an opposite change. These findings further verified the negative regulation of VdRim15 by these epigenetic factors, and demonstrated that a dynamic regulation of epigenetic homeostasis of 5-mC and H3K9me3 is required for the preparation of *V. dahliae* infection. Therefore, identification of the inducible mechanism underlying these dynamic changes in epigenetic modifications, during infection, is worthy of further exploration in future studies.

## Methods

### Fungal strain and culture medium

Virulent defoliating *V. dahliae* strain V592 from cotton was used in this study. The PDA medium, Czapek–Dox Medium, CM medium and IM medium were used for fungal phenotype observation, spore or mycelium collection and *Agrobacterium* mediated transformation (ATMT). The medium was prepared as previously described (Gao et al. 2010).

### Plant growth condition

*Arabidopsis* plants were grown on soil at 22 °C with relative 70% humidity. Cotton, plants were grown on soil at 25 °C with relative 70% humidity. All plants were grown under a 16 h light/8 h dark photoperiod.

### Plasmid constructs and fungal transformation

For gene knockout constructs, 1 kb genomic sequences of both upstream and downstream of gene were amplified and the resulted PCR products were ligated to *pGKO-HYG* plasmid simultaneously with ClonExpress MultiS One Step Cloning Kit (Vazyme, C113). Homologous recombination was conducted according to previous report (Wang et al. 2016). For gene over-expression and complementation constructs, coding sequence was ligated into *Tef-Flag* vector with ClonExpress II One Step Cloning Kit (Vazyme, C112). All constructs were transformed into *Agrobacterium tumefaciens* strain EHA105. The *Agrobacterium tumefaciens*-mediated transformation method (Frandsen 2011) was used for gene deletion, gene complementation and gene over-expression in V592 strain. Primers used for plasmid construction and fungal transformation were listed in Table S1.

### Fungal phenotype observation

For fungal phenotype observation, single colony separation of V592 and all transformed strains were achieved by spreading each corresponding spore suspension of 10^2^–10^3^ spore mL^−1^ on Czapek–Dox Medium for 2 days culture at 25 °C in the dark. Then each single colony of each strain was inoculated on PDA medium for another 14-days culture. The pictures of each fungal strain phenotype were taken (at least 3 individual colony for each fungal strain). Then each fungal strain colony and microsclerotia diameters were measured with ruler.

### Fungal inoculation and pathogenicity assays

The conidia of *V. dahliae* strains were collected and re-suspended at a concentration of 10^6^ mL^−1^ and used for plant inoculation (Qin et al. 2018). *Arabidopsis* and cotton, were infected by the root-dipping inoculation method described previously (Ellendorff et al. 2009; Qin et al. 2018). After visible symptom development post-inoculation, *Arabidopsis* rosette leaves and cotton stems were harvested and flash-frozen in liquid nitrogen. Each sample (100 mg) was isolated for DNA using DNAsecure Plant Kit (TIANGEN, DP320), which was quantified for fungal biomass through RT-qPCR method. (Ellendorff et al. 2009).

### 5-Aza treatment

The single colony separation of V592 strain was prepared as described in “Fungal phenotype observation” part. 5’-Aza powder was dissolved in DMSO and added to PDA medium to make final concentration of 0.5 µM (the control medium was added with same amount of DMSO). The infection phenotype observation and fungal biomass detection were applied as described earlier.

### 5-mC Dot blot

5-mC dot blot was performed as previously described with minor modifications (Jia et al. 2017). In brief, gDNA extracted from mycelium was denatured and spotted on N + membrane (Cytiva, RPN303B). After UV crosslinking (AnalytikJena, CL-1000L) and blocking, membrane was incubated with 5-mC mouse monoclonal antibody (1:1000 dilution) (EPIGENTEK, A-1014) overnight at 4 °C. After sequential washing, membrane was incubated with HRP secondary antibody (1:10,000 dilution) (abmart, M21001) for 1 h at room temperature and detected by ECL western blotting reagent (Beyotime, P0018FS).

### RNA isolation and RT-qPCR

Total RNA from mycelium was isolated with the RNAprep Pure Plant Kit (TIANGEN, DP441). Then total RNA was used for *TransScript* One-Step gDNA Removal and cDNA Synthesis SuperMix (TransGen Biotech, AT311). Quantitative PCR was performed with *PerfectStart* TM Green qPCR SuperMix (TransGen Biotech, AQ601) following manufacture’s protocol. For each reaction three biological replicates were used. Primers used for qPCR were listed in Table S1.

### Whole-genome bisulfite sequencing

V592 strain and transformed strains conidia suspension were cultured in CM liquid medium at 120 rpm for 3 days. The mycelia were collected for genomic DNA extraction through filtration. The genomic DNA was extracted by using DNAsecure Plant Kit (TIANGEN, DP320). Bisulfite conversion, library construction, and deep sequencing were performed by the NovogeneCo. (Beijing, China). Briefly, Next generation sequencing library preparations were constructed following the manufacturer’s protocol (NEBNext® Ultra™ DNA Library Prep Kit for Illumina®). For each sample, 1 μg genomic DNA was randomly fragmented to < 500 bp by sonication (Covaris S220). The fragments were treated with End Prep Enzyme Mix for end repairing, 5′ Phosphorylation and dA-tailing in one reaction, followed by a T-A ligation to add Methylated adaptors to both ends. Size selection of Adaptor-ligated DNA was then performed using AxyPrep Mag PCR Clean-up (Axygen), and fragments of ~ 410 bp (with the approximate insert size of 350 bp) were recovered. Then bisulfite conversion was performed using EZ DNA Methylation-Lightning™ Kit (Zymo Research). Each sample was then amplified by PCR for 14 cycles using P5 and P7 primers, with both primers carrying sequences which can anneal with flow cell to perform bridge PCR and P7 primer carrying a six-base index allowing for multiplexing. The PCR products were cleaned up using AxyPrep Mag PCR Clean-up (Axygen), validated using an Agilent 2100 Bioanalyzer (Agilent Technologies, Palo Alto, CA, USA), and quantified by Qubit2.0 Fluorometer (Invitrogen, Carlsbad, CA, USA). Then libraries with different indices were multiplexed and loaded on an Illumina HiSeq instrument according to manufacturer’s instructions (Illumina, San Diego, CA, USA). Sequencing was carried out using a 2 × 150 paired-end (PE) configuration; image analysis and base calling were conducted by the HiSeq Control Software (HCS) + OLB + GAPipeline-1.6 (Illumina) on the HiSeq instrument.

### Fungal penetration assay

Sterilized cellophane membrane (Solarbio, YA0620) was overlaid onto MM medium. Spores (3 × 10^5^ spore mL^−1^) collected from liquid culture of each certain strain were striped on MM medium with cellophane for 3-day culture at 28 °C. Then cellophane was removed from MM medium which was cultured for another 5-day. The photos of each strain were taken at least from 3 individual plates.

### Yeast two-hybrid and genetic transformation

Y2H assay was performed as previously described (Chang et al. 2022). In brief, cDNAs of DIM2 and HP1 were amplified from V592 and JR2 cDNA and fused into the GAL4 activation domain vector (pGADT7) and the GAL4 binding domain vector (pGBKT7), respectively. Two constructs were co‐transformed into yeast strain AH109 and transformants were selected by growth on selective dropout medium SD−LWA (lacking Leu, Trp, Ade). As yeast transformation, for gene knockout construction, 500 bp genomic flank sequences of both upstream and downstream of gene were amplified and fusion with screening sequence. PCR product was transformed into yeast strain BY4742 and transformants were selected by growth on selective dropout medium SD-L (lacking Leu). For gene over-expression, cDNA of VdRIM15 was amplified from V592 cDNA and fused into pYES2 vector. Construct was transformed into yeast mutant and transformants were selected by growth on selective dropout medium SD−LU (lacking Leu, Ura). Primers used for Y2H and transformation were listed in Table S1.

### Stress treatment

For *V. dahliae* treatment, spore suspension of V592 and all transformed strains were inoculated on PDA medium with H_2_O_2_, NaCl and high pH, respectively. Subsequent culture and data collection were the same as fungal phenotype observation. Yeast stress treatment was performed as previously described (Kim 2020). In brief, yeast WT strain and transformants were cultured on both YPDA agar and SC minimal medium including Pb(NO_3_)_2_ (Sigma, 203580) or CdCl_2_ (Sigma, 655198)at 28 °C for 6 days, respectively.

### Ca^2+^ and ROS detection

Cytoplasmic Ca^2+^ detection was performed as previously described (Zhao et al. 2016). In brief, a stock solution of Fluo-4AM (MedChemExpress, HY-101896), 4 mM in dimethyl sulfoxide (DMSO), was prepared and diluted with double distilled water to make a 5 μM work solution. Mycelium grown on cellophane for 2 days was loaded with 5 μM Fluo-4AM for 30 min at 28 °C, washed three times and GFP signal was captured with 488-nm lasers by SR5 (Leica, Germany). For ROS detection, cellophane with mycelium at 2 dpi was stained by DCFH-DA (1:1000 dilution) (Beyotime, S0033S) for 20 min at 37 °C, washed three times and GFP signal was captured with 488-nm lasers by SR5 (Leica, Germany).

### Bisulfite PCR

Bisulfite PCR was performed as previously described (Guo et al. 2022). In brief, mycelium was collected for DNA extraction and 1 µg DNA was using for bisulfite conversion by EZ DNA Methylation Kit (Zymo Research, D5001). Unbiased amplification for conversed DNA was performed, then the PCR products were cloned into pMD18-T vectors (Takara, 6011) and individual clones were sequenced by Sanger sequencing. Bisulfite primer information is presented in Table S1.

### In vitro phosphorylation assay

In vitro phosphorylation assay was performed as previously described (Chen et al. 2022). In brief, recombinant GST–VdRIM15 was incubated with GST-tagged VdMSN2 in 20 μL of reaction buffer [50 mM Tris–HCl, pH 7.0, 20 mM MgCl_2_, 1.5 mM ATPγS (Abcam, ab138911)] at room temperature for 30 min. Then, 2.5 mM PNBM (Abcam, ab138910) in DMSO was added. After incubation for 1 h, the proteins were separated by SDS–PAGE. After electrophoresis, the gel was transferred to a PVDF membrane for western blots. Anti-thiophosphate ester antibody (Abcam, ab92570) was used to detect phosphorylated proteins.

### Chromatin immunoprecipitation

ChIP assay was performed as previously described with minor modifications (Zhang et al. 2023). In briefly, 2 g of *V. dahliae* mycelium was collected and grinded in liquid nitrogen and crosslinked in nuclei isolation buffer with 1% formaldehyde for 15 min at room temperature. After stopping crosslink, filtering, centrifuge and washing, the nucleus pellet was resuspended with 500 µL of nuclear lysis buffer. The lysates were diluted twofold with dilution buffer and sheared by sonication. After centrifugation, the supernatant was diluted fivefold with dilution buffer and incubated with 5 µL of H3K9me3 antibody (Abcam, ab195412) and 50 µL of Dynabeads protein G (Invitrogen, 10004D) overnight at 4 °C. After sequential washing, the DNA–protein complex was eluted and cross-linking was reversed at 65 °C for 7 h. After proteinase K and RNaseA treatment, the recovered DNA was subjected to qPCR analysis. ChIP-qPCR primer information is presented in Table S1.

### Supplementary Information

Below is the link to the electronic supplementary material.

Supplementary file1 (DOCX 6182 KB)

## Data Availability

The whole genome bisulfite sequencing data has been deposited in NCBI SRA with the bioproject number PRJNA922710.
