# Supplementary material for: DNA methylation-dependent epigenetic regulation of Verticillium dahliae virulence in plants
Source: aBIOTECH. 2023 Sep 20;4(3):185–201. doi: 10.1007/s42994-023-00117-5 (PMC10638132; doi:10.1007/s42994-023-00117-5)
Supplement: Supplementary file 1 — Supplementary file1 (DOCX 6182 KB) [file 42994_2023_117_MOESM1_ESM.docx]

**Supplementary Figures and Tables**

**Supplementary Figures**


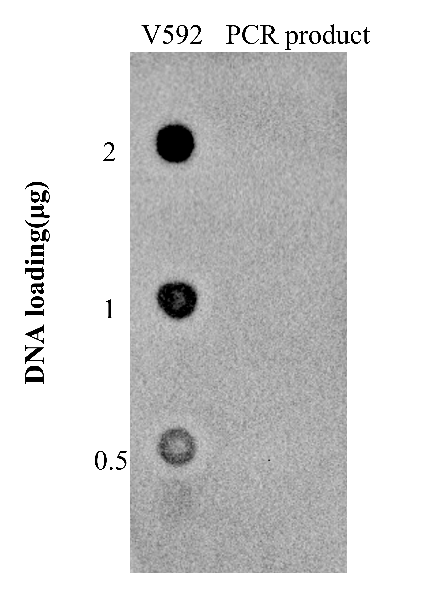


**Figure. S1: 5-mC-specific dot blot assay of *Verticillium dahliae*.** 2 μg、1 μg and 0.5 μg of gDNA were loaded per dot. The PCR product was used as negative control.


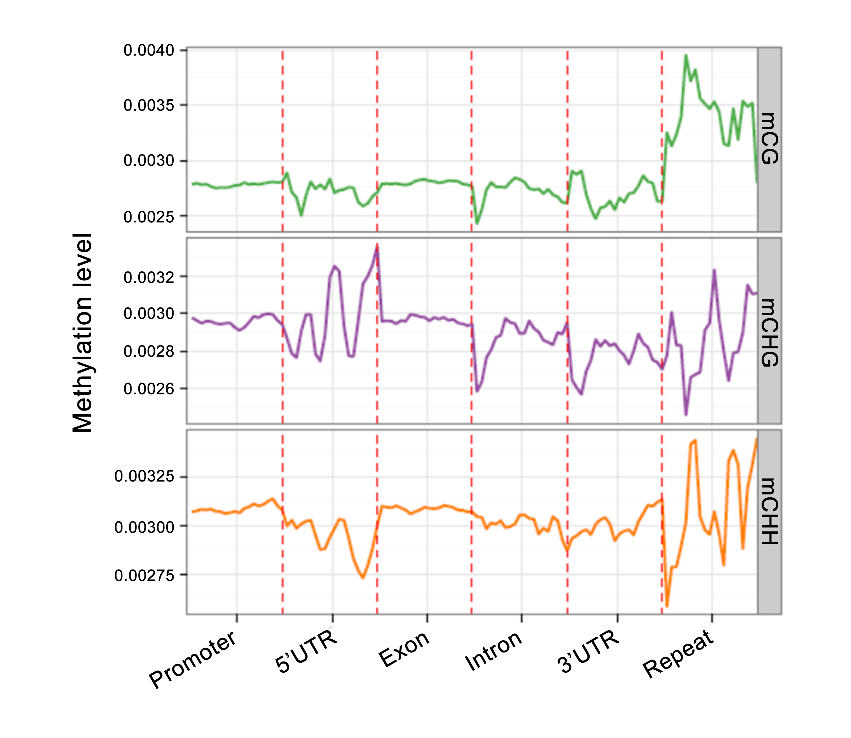


**Figure. S2: Distribution of DNA methylation in different regions of *Verticillium dahliae* V592 genome.**


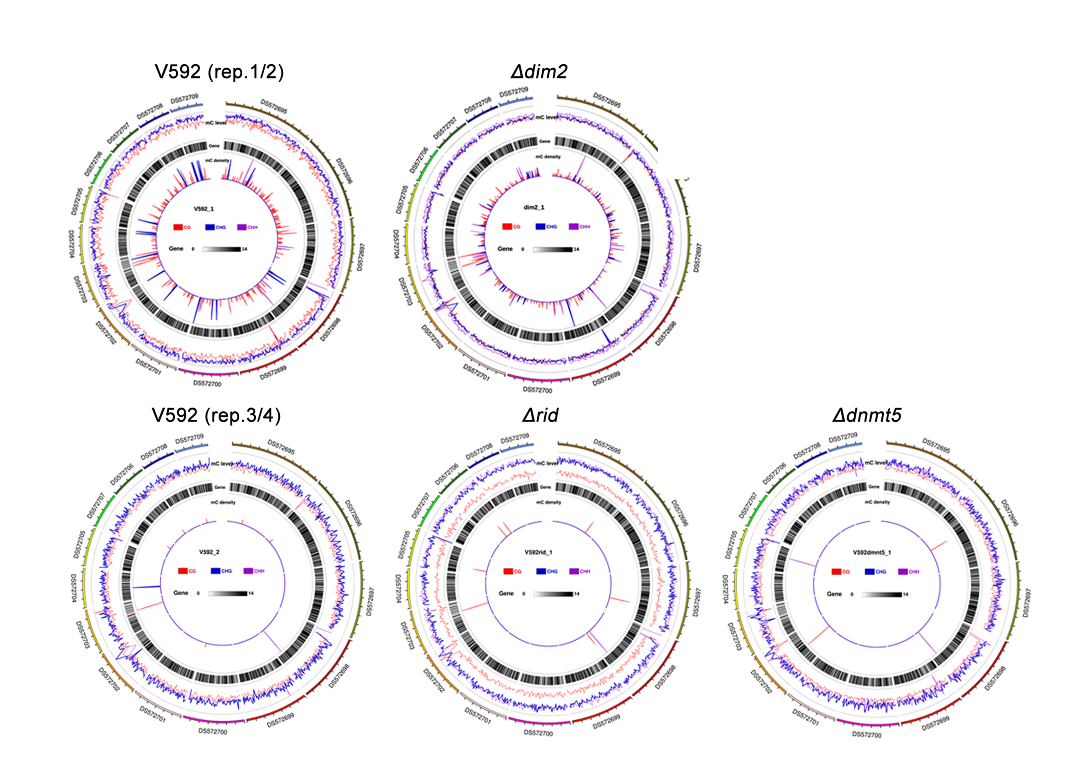


**Figure. S3: Circos diagrams of DNA methylation density level of chromosome.** DNA methylation density level of V592(rep.1/2), Vd*Δdim2*, V592(rep.3/4), Vd*Δrid* and Vd*Δdnm5*.


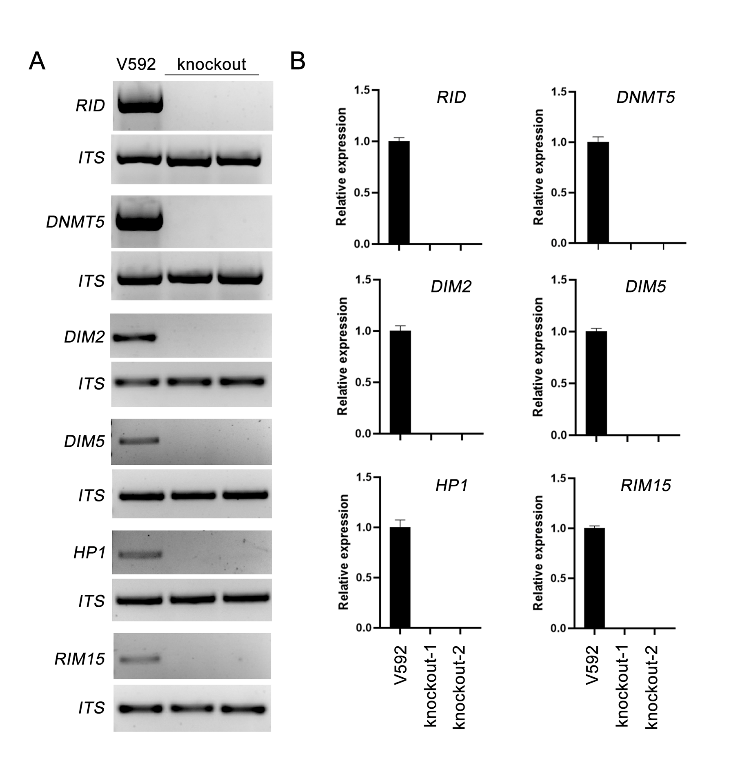


**Fig. S4 Analysis of targeted gene deletion mutants**. **A**: Genomic DNA extracted from V592 wild type strain and putative of Vd*Δrid,* Vd*Δdnmt5*, Vd*Δdim5*, Vd*Δhp1* and Vd*Δrim15* transformants were used for PCR detection. ITS serves as control. **B**. Reverse transcription qPCR (RT-qPCR) analysis of the expression of Vd*RID*, Vd*DNMT5*, Vd*DIM5*, Vd*HP1* and Vd*RIM15* in knockout mutants with primers listed in TableS1.


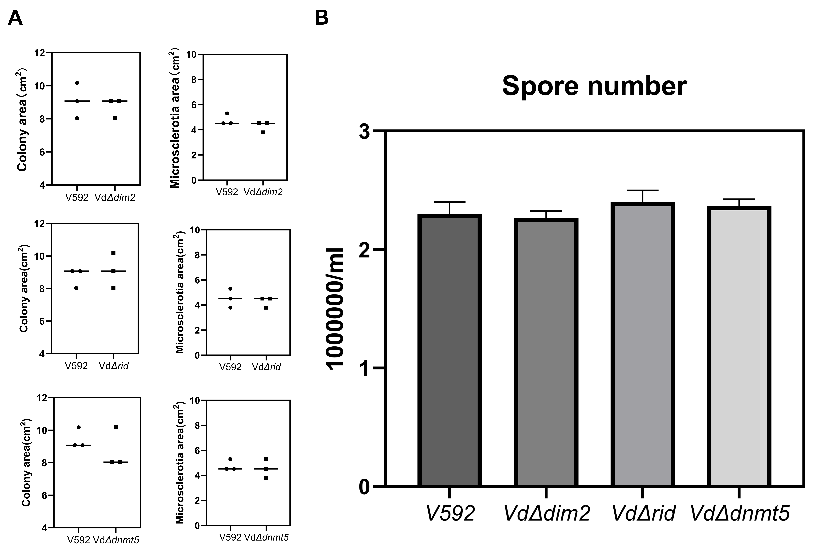


**Fig. S5 Developmental phenotypes of V592, Vd*Δdim2*, Vd*Δrid* and Vd*Δdnmt5* mutant strains**. **A.** Diagrams showing the colony (left panel) and microsclerotia (right panel) areas of V592, Vd*Δdim2*, Vd*Δrid* and Vd*Δdnmt5* mutants. Data were collected 14 days after culture, the scatter in the diagram represents three biological replications. **B.** Column diagrams showing the spore number of V592, Vd*Δdim2*, Vd*Δrid* and Vd*Δdnmt5* mutants. Data are the mean ± SD from three biological replicates.


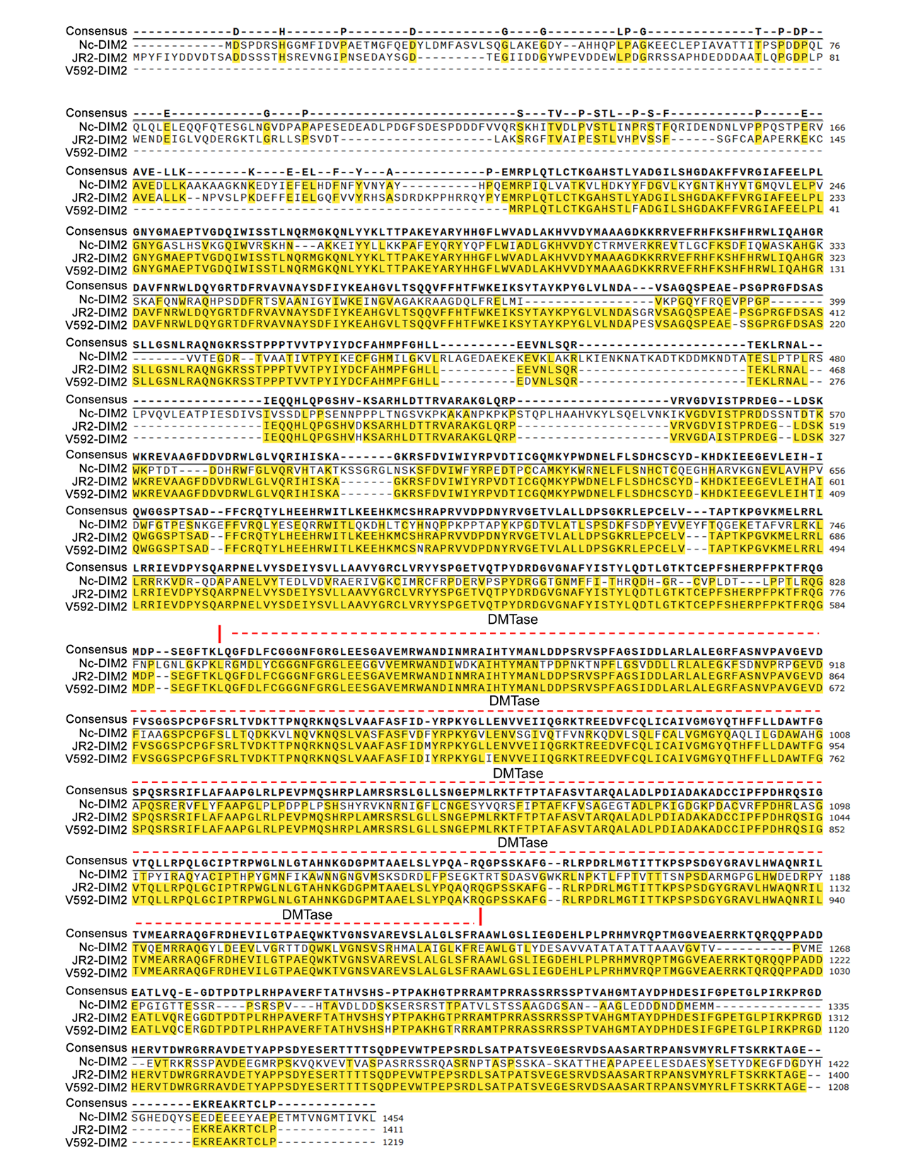


**Fig. S6 Sequence alignment of the amino acids of DIM2 proteins from *N. crassa*, *V. dahliae* JR2 and V592 strains.** Protein sequence alignment of full length from NcDim2, VdDim2 in JR2 and VdDim2 in V592. The DMTase region was marked with red dotted line.


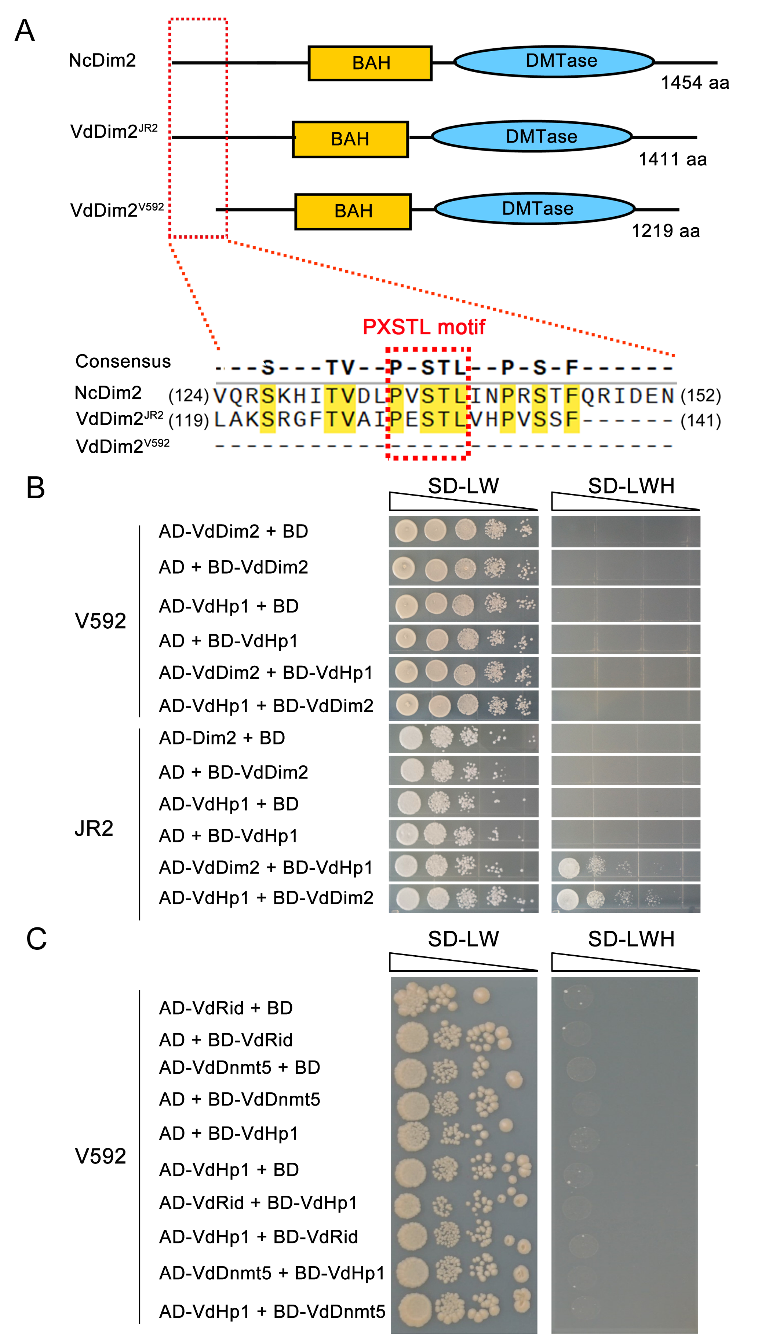


**Fig. S7 Natural variation in the N-terminal region results in the disability of VdDim2^V592^ interaction with VdHp1.**

**A**. Upper panel, comparison of DIM2 protein domain structure in *N. crassa*, JR2 and V592, respectively. Lower panel, protein sequence alignment of the N-terminal regions from NcDim2, VdDim2 in JR2 and VdDim2 in V592. The PXSTL-like motif was marked with red dotted line. **B**. Y2H results showing the interaction between VdHp1 and VdDim2 proteins from JR2 and V592 strains. **C.** Y2H results indicate that VdHP1 does not interact with VdRid and VdDnmt5 in V592 strains.


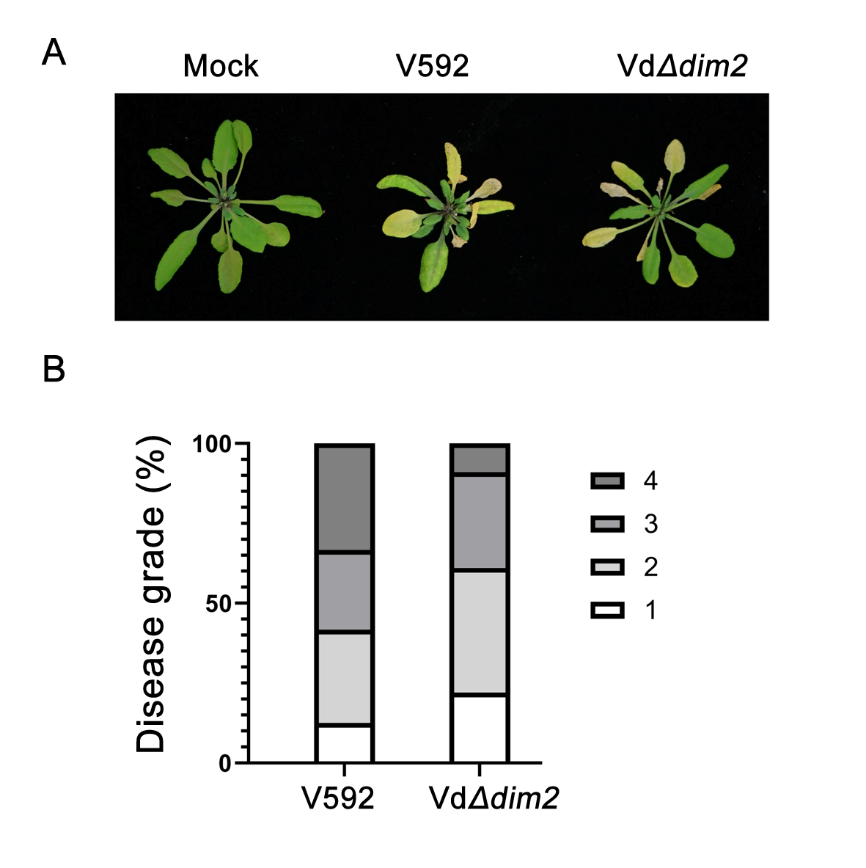


**Fig. S8 VdDim2 dysfunction leads to weak reduction of fungal virulence.**

**A.** The disease symptoms of *A. thaliana* plants (Col-0) inoculated with V592 and VdΔdim2 strains. Photographs were taken at 21 dpi. Mock represents buffer-inoculated plant controls. B. The disease grade of inoculated plants.


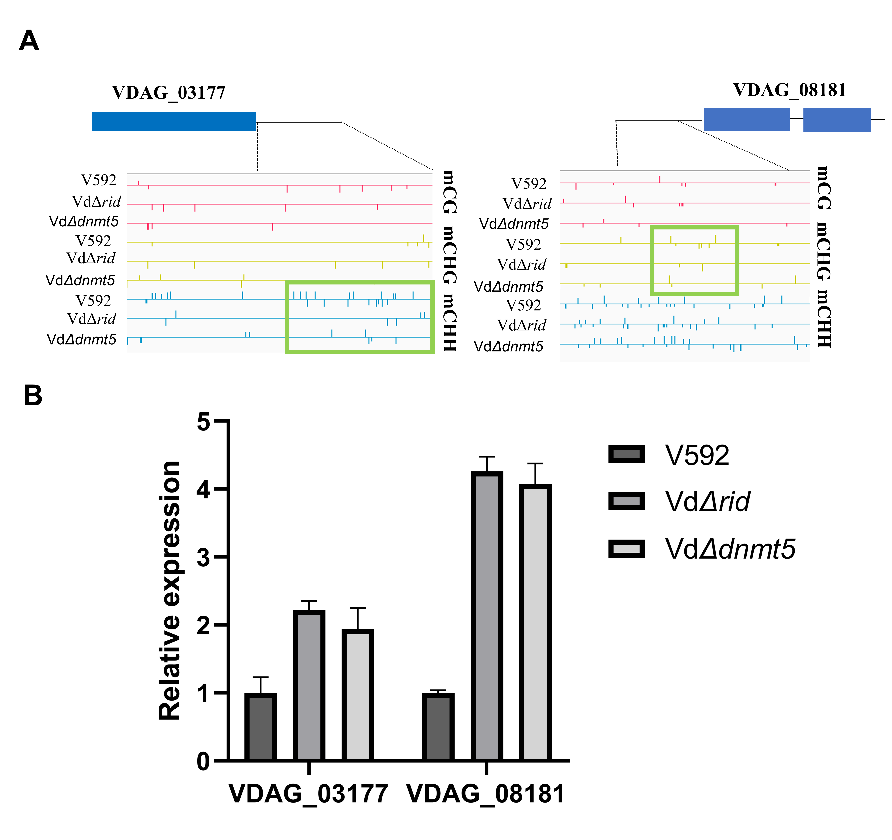


**Fig. S9 DNA methylation and gene expression of *Vd03177* and *Vd08181* pattern in V592, Vd*Δrid*, and Vd*Δdnmt5* strains. A.** Snapshot of WGBS showing the DNA methylation levels at *Vd03177* and *Vd08181* promoter region in V592, Vd*Δrid*, and Vd*Δdnmt5* strains. One representative result of two WGBS replicates was shown for each genotype. **B.** RT-qPCR results showing the relative *Vd03177* and *Vd08181* RNA levels in V592, Vd*Δrid*, and Vd*Δdnmt5* strains. Data are the mean ± SD from three biological replicates.


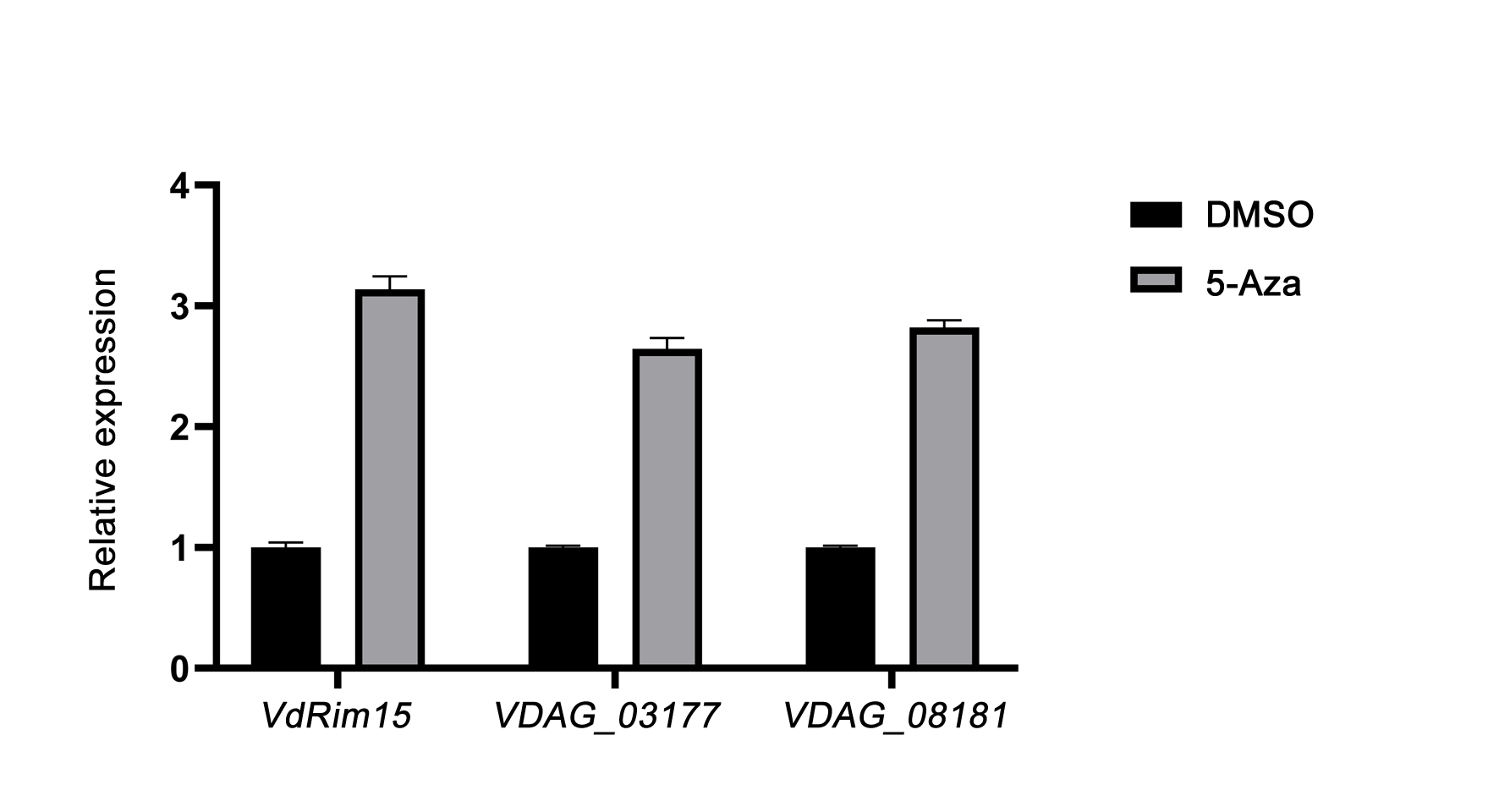


**Fig. S10 5-Aza treatment increases the expression of representative VdRid and VdDnmt5 target genes.**

RT-qPCR results showing the relative *VdRim15*, *Vd03177* and *Vd08181* RNA levels in DMSO- and 5-Aza-treated V592 strains. Data are the mean ± SD from three biological replicates.


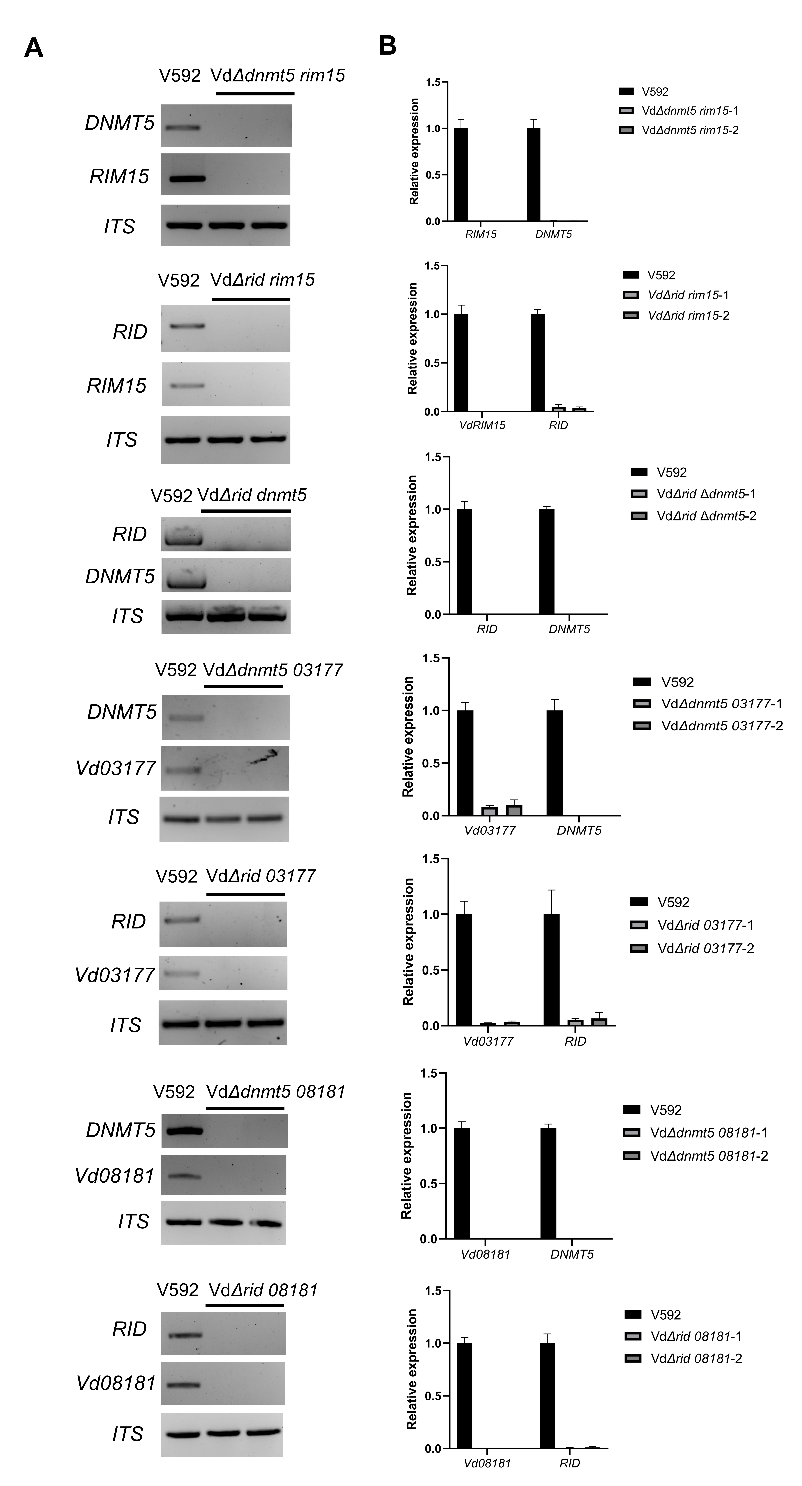


**Fig. S11 Identification of targeted gene deletion in double mutants**. **A**: Genomic DNA of V592 wild type strain and putative transformants were used for PCR detection. ITS serves as control. **B**. Reverse transcription qPCR (RT-qPCR) analysis of the expression of target genes in knockout mutants with primers listed in Table S1.


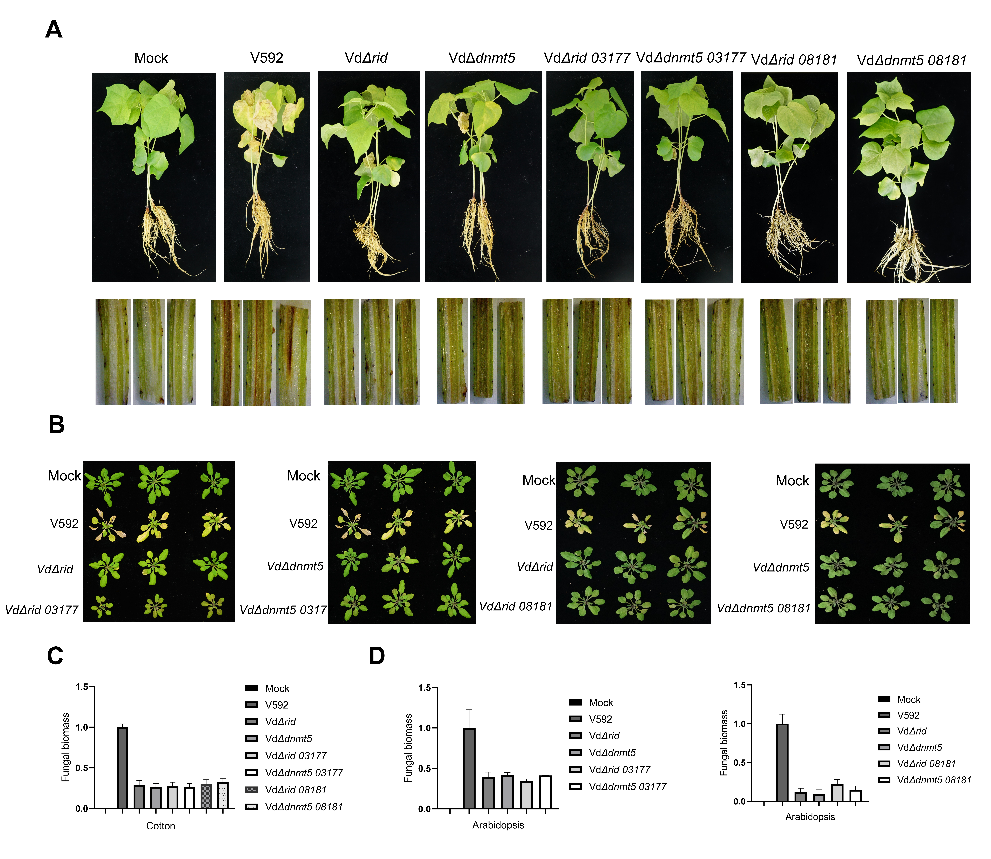


**Fig. S12 *Vd03177* and *Vd08181* do not participate in *VdRID-* and *VdDNMT5*-mediated virulence regulation. A.** The disease symptoms of cotton plants infected with V592 and mutant strains at 21 dpi. The longitudinal section of the stem from inoculated plants were shown. **B.** The disease symptoms of *A. thaliana* plants infected with V592 and mutant strains at 21 dpi. **C-D**. Relative fungal biomass analysis of cotton and *A. thaliana* plants infected with V592 and mutant strains at 21dpi.


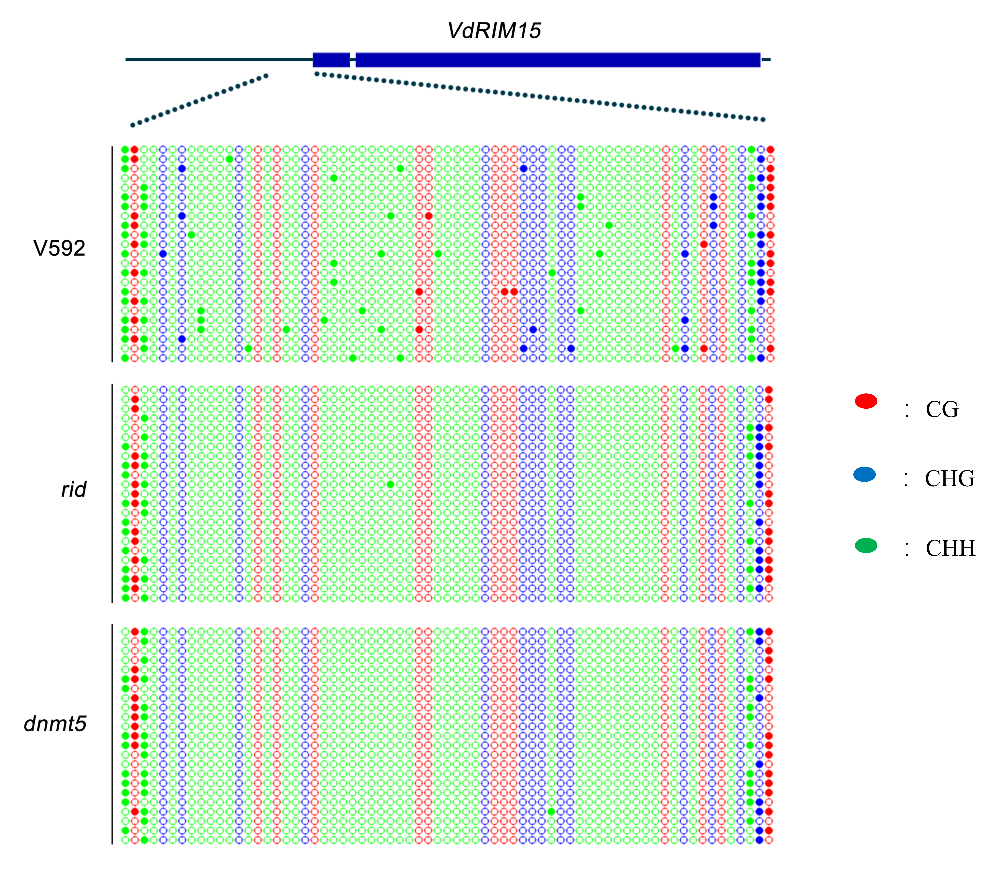


**Fig. S13 Individual bisulfite sequencing results of *VdRim15* promoter.** Individual bisulfite sequencing results of *VdRIM15* promoter in V592, Vd*Δrid* and Vd*Δdnmt5*. The red, blue and green solid cycles indicate CG, CHG and CHH methylation, respectively.


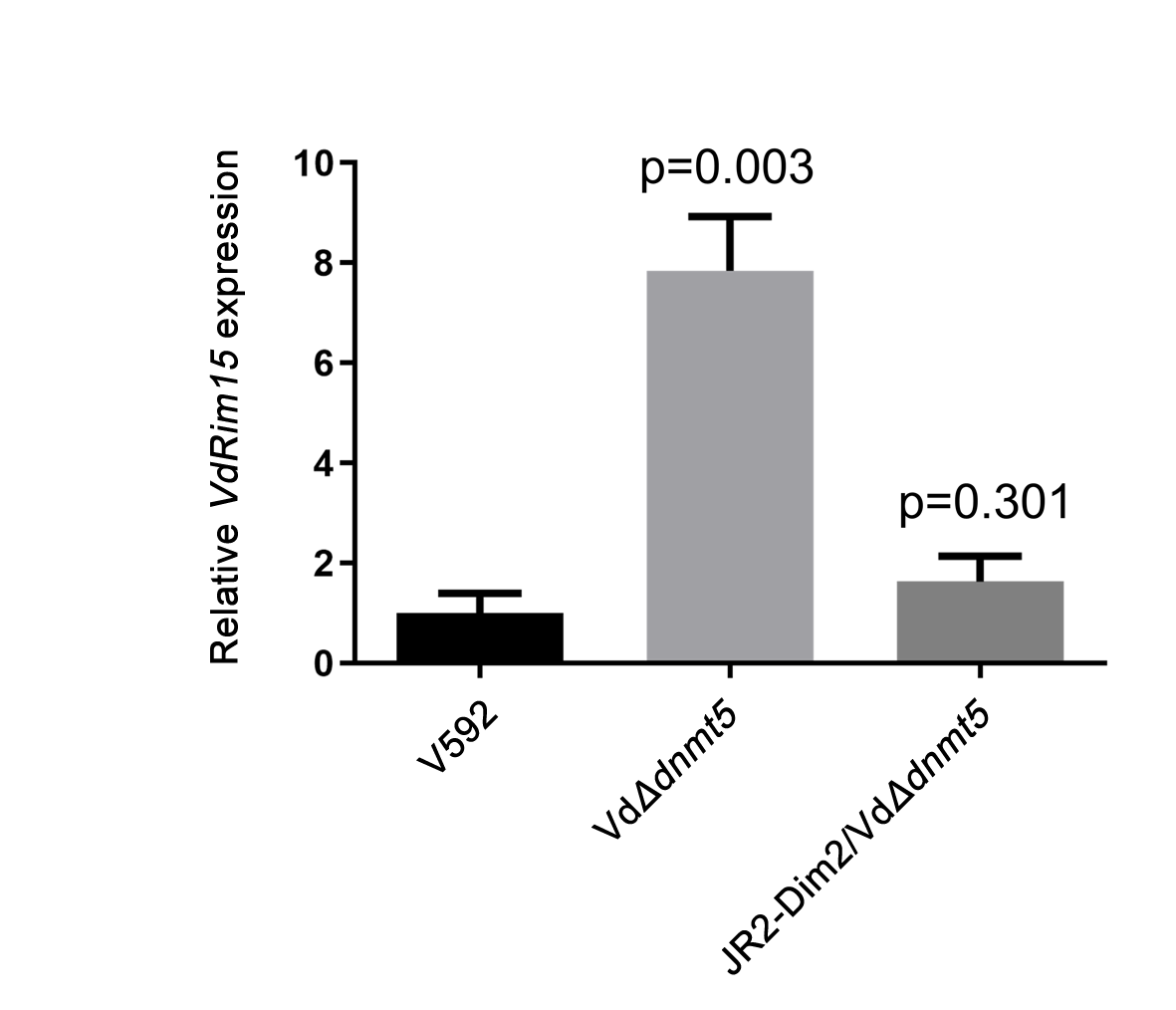


**Fig. S14 JR2-Dim2 can rescue the up-regulation of VdRim15 expression in Vd***Δ****dnmt5*.**

RT-qPCR results showing the relative *VdRim15* RNA levels in V592, Vd*Δdnmt5* and JR2-Dim2/ Vd*Δdnmt5* strains. Data are the mean ± SD from three biological replicates.

**
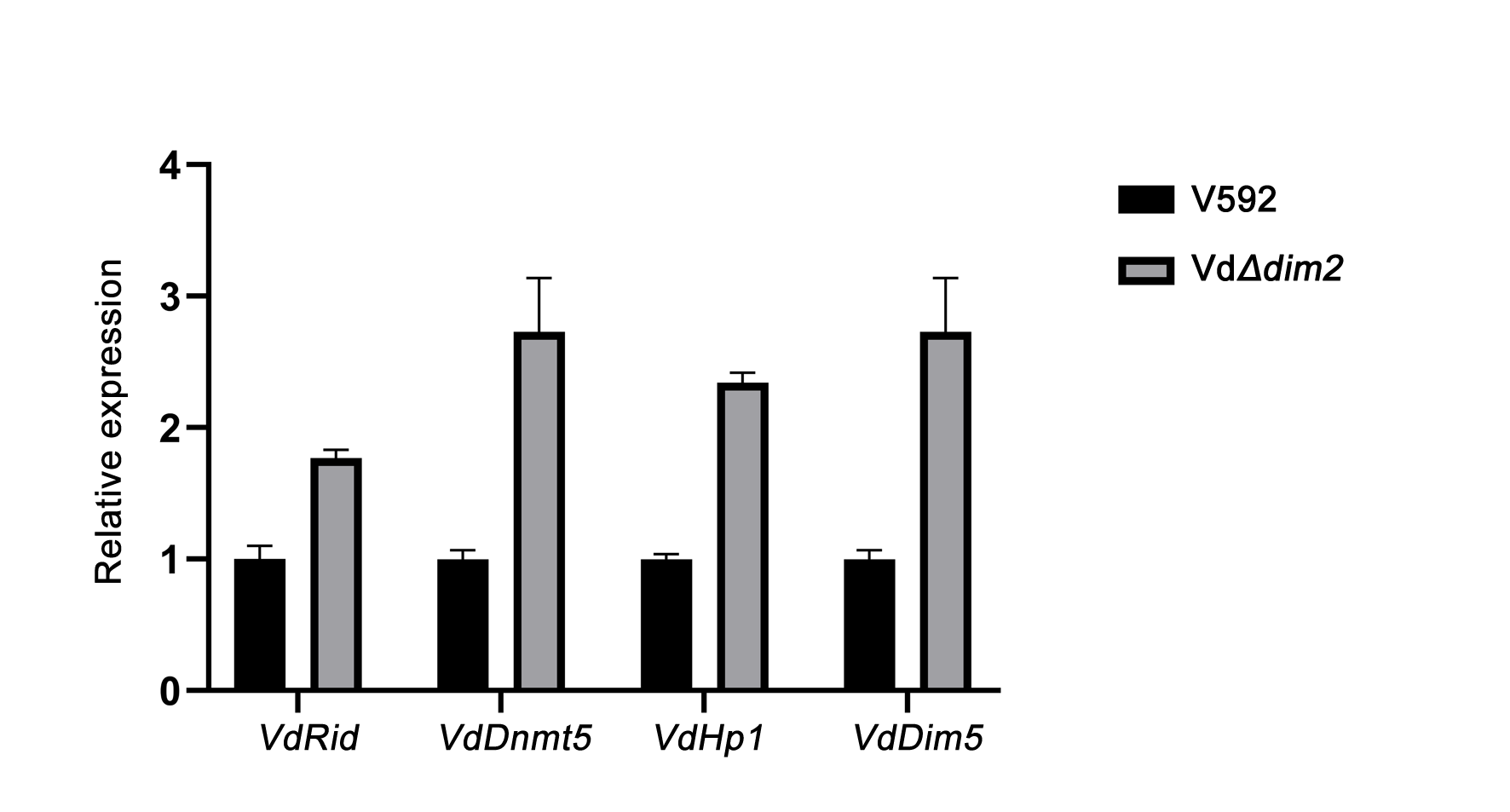
**

**Fig. S15 The expression of DNA and H3K9 methyltransferase genes in V592 and Vd***Δr****dim2*.**

RT-qPCR results showing the relative *VdRid*, *VdDnmt5*, *VdHp1* and *VdDim5* RNA levels in V592 and Vd*Δdim2* strains. Data are the mean ± SD from three biological replicates.

**
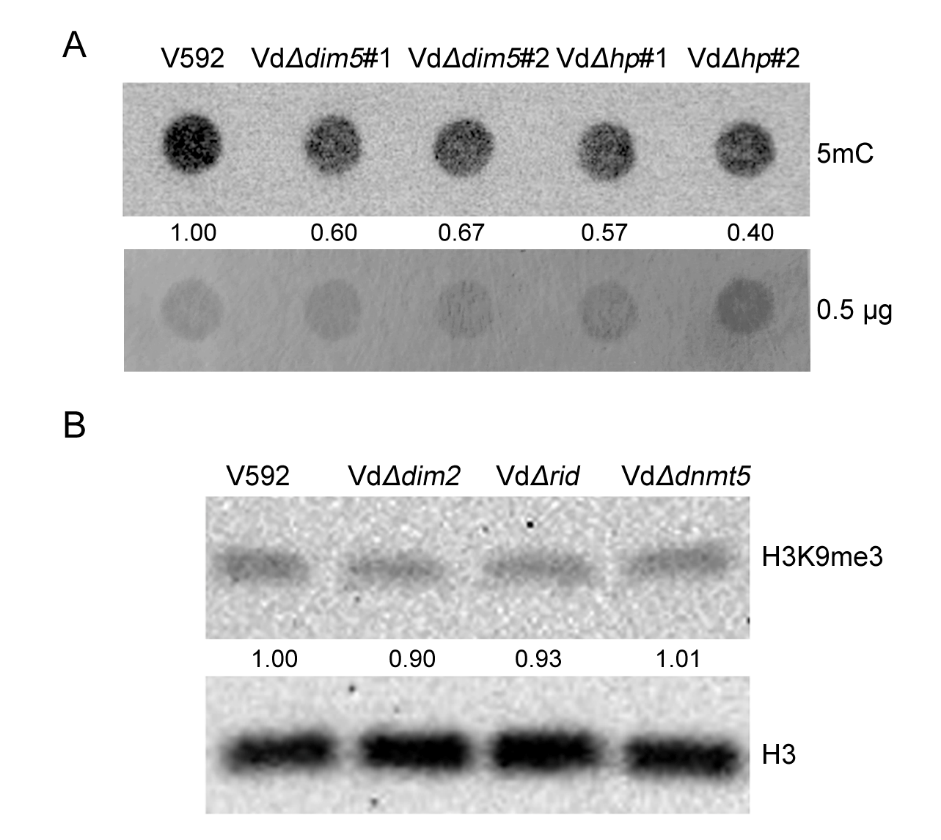
**

**Fig. S16 DNA methylation and H3K9me3 levels in different strains.**

A. 5-mC dot blot results showing the obvious reduction of 5-mC levels inV592, Vd*Δdim5* and *VdΔhp1* strains. B. Western blotting results showing the H3K9me3 levels in V592, Vd*Δdim2*, *VdΔrid* and *VdΔdnmt5* strains*.*

**Supplementary Tables**

**Table S1. List of primers used in this study**

| DIM2（JR2）-AD-F | GTACCAGATTACGCTCATATGatgccgtacttcatatatgatgacg |
| --- | --- |
| DIM2（JR2）-AD-R | ATGCCCACCCGGGTGGAATTCctagggcagacatgtcctttttgct |
| DIM2（JR2）-BD-F | TCAGAGGAGGACCTGCATATGatgccgtacttcatatatgatgacg |
| DIM2（JR2）-BD-R | TCGACGGATCCCCGGGAATTCctagggcagacatgtcctttttgct |
| DIM2（V592）-AD-F | GTACCAGATTACGCTCATATGatgcgcccgcttcagacactgtg |
| DIM2（V592）-AD-R | ATGCCCACCCGGGTGGAATTCctagggcagacatgtcctttttgct |
| DIM2（V592）-BD-F | TCAGAGGAGGACCTGCATATGatgcgcccgcttcagacactgtg |
| DIM2（V592）-BD-R | TCGACGGATCCCCGGGAATTCctagggcagacatgtcctttttgct |
| HP1-AD-F | GTACCAGATTACGCTCATATGATGCCGCCAGCACTCAGCGACA |
| HP1-AD-R | ATGCCCACCCGGGTGGAATTCTTATTTGAGCTCGGCATCCGTCGTG |
| HP1-BD-F | TCAGAGGAGGACCTGCATATGATGCCGCCAGCACTCAGCGACA |
| HP1-BD-R | TCGACGGATCCCCGGGAATTCTTATTTGAGCTCGGCATCCGTCGTG |
| RIM15-BSP-F | AGAAGGGAAAGYAAAGYGAAAYAAAG |
| RIM15-BSP-R | CAATCCTCCRTACCCATACCARATRCA |
| ITS-F | AAAGTTTTAATGGTTCGCTAAGA |
| ITS-R | CTTGGTCATTTAGAGGAAGTAA |
| AtRubisco-F | GCAAGTGTTGGGTTCAAAGCTGGTG |
| AtRubisco-R | CCAGGTTGAGGAGTTACTCGGAATGCTG |
| ELF-qF | CCATTGATATCGCACTGTGG |
| ELF-qR | tggagataccagcctcgaac |
| VdGAPDH-F | CGAGTCCACTGGTGTCTTCA |
| VdGAPDH-R | CCCTCAACGATGGTGAACTT |
| CottonH3-F | CGGTGGTGTGAAGAAGCCTCAT |
| CottonH3-R | AATTTCACGAACAAGCCTCTGGAA |
| DIM5-qF | GTGCCAGATTCGAGCATTTC |
| DIM5-qR | ACTGGAGGAACCGAAAGTTG |
| VDAG_08181-qF | CTCGATGTCCTCAACAAGCA |
| VDAG_08181-qR | GGCAATGGGAGAGAAGAGAAG |
| RIM15-qF | AATCCCAGTCAAACCTGGATAC |
| RIM15-qR | CGTCTCAGGCGCAAGATAAT |
| VDAG_03177-qF | TTCGTCCACGATGACTACCT |
| VDAG_03177-qR | TGGCGGCCTCCTCTATG |
| RID-qF | CAACCAGCGATATACCCTCTTC |
| RID-qR | CAAGCCTTATCCCATACGTCTAC |
| DNMT5-qF | GTCCAAGATCTCCTCGACATTAC |
| DNMT5-qR | AAGACGCACCCGACAATAC |
| DIM2-qF | AATGGAGCTTCGGAGACTATTG |
| DIM2-qR | CCAGCAGGACGCTGTAAAT |
| HP1-qF | TCCAGAACACCGAACAAGTC |
| HP1-qR | CGTCTTCATCGGCATCATCT |
| NoxB-qF | GATGTTGCCCTCGTACTCTTT |
| NoxB-qR | CGTCTTGTGGAAGGTGATGT |
| Pls1-qF | GTCACCTCGTACTCCAACATAC |
| Pls1-qR | GTAGCACAGCAAGGCAGATA |
| Crz1-s | tttactcgcgcgtacaacct |
| Crz1-a | aacttcttctcgcccgagtg |
| RIM15-ChIP-F1 | AGACTTCTGGCTACAACATCCCCG |
| RIM15-ChIP-R1 | ATTTTGCAGGGTCGTCGATACCATT |
| RIM15-ChIP-F2 | ACGGTTCTTCTCTTCTTCACTTCGG |
| RIM15-ChIP-R2 | TCCGAGACAGAAGACAGTCGCTGA |
| pGKO-DIM2-UP-F | tgaggtcttaattaaGTTAACATGGATTATGCCCAGGAGATCTCGACG |
| pGKO-DIM2-UP-R | actagtgctgaggcaCAATTGCGTTCATTAAGATGAGTGAGCCATGAG |
| pGKO-DIM2-Dn-F | gaattcactggccgcCACGTGACGTCTGGGTGTGGAAGAATCCTT |
| pGKO-DIM2-Dn-R | TAGAACTAGTGGATCCCCGGGCGGTCCCCAACCCCGGACAGGCC |
| pGKO-HP1-UP-F | tgaggtcttaattaaGTTAACGTGAGATCAGCTGGAGGAGATGCC |
| pGKO-HP1-UP-R | actagtgctgaggcaCAATTGCTTGATGGAAGAGTGGAAGCAGGAA |
| pGKO-HP1-Dn-F | gaattcactggccgcCACGTGTAAGTGCACAGTTTACGAAGAGCTATGACA |
| pGKO-HP1-Dn-R | TAGAACTAGTGGATCCCCGGGAGGTGTGGTGTATGTGGGTGTCTCA |
| pGKO-DIM5-UP-F | tgaggtcttaattaaGTTAACTGTGCCTTTGAGGGCGTCTGCGA |
| pGKO-DIM5-UP-R | actagtgctgaggcaCAATTGGTTGACTGATCTGTAAATAAGAAATGACAC |
| pGKO-DIM5-Dn-F | gaattcactggccgcCACGTGCGAGGCACCGGATGGGAGGGGC |
| pGKO-DIM5-Dn-R | TAGAACTAGTGGATCCCCGGGGCCTGTGTGGAGGCGGCTGCGGT |
| pGKO-RID-UP-F | tgaggtcttaattaaGTTAACACATTCTCGTACAATGATTAAATGCCCGT |
| pGKO-RID-UP-R | actagtgctgaggcaCAATTGTCTACAATGTCGCCCACTCCACGG |
| pGKO-RID-Dn-F | gaattcactggccgcCACGTGAGAGGGGACGATGAAGTGTTGCTTGCCGTG |
| pGKO-RID-Dn-R | TAGAACTAGTGGATCCCCGGGTCGCCCGCCCAGCTCGCCCCCATGTAC |
| pGKO-DNMT5-UP-F | tgaggtcttaattaaGTTAACCGTGCTGATAGCCACCAACCTAATC |
| pGKO-DNMT5-UP-R | actagtgctgaggcaCAATTGAGTGATGGTGGTTGCGATGGTGGT |
| pGKO-DNMT5-Dn-F | gaattcactggccgcCACGTGGATGGGCAACATCAAGCACGTGGAA |
| pGKO-DNMT5-Dn-R | TAGAACTAGTGGATCCCCGGGACACGCTTCTGCTGAACCGCCCCT |
| 1300-RID-Flag-F | atttggagaggacagggtaccatggcttccccctacgggatctct |
| 1300-RID-Flag-R | agtgtcgactctagaggatccttgccactccatgggcggcctgta |
| 1300-DNMT5-Flag-F | atttggagaggacagggtaccatggctcagaaaccccagcaattcg |
| 1300-DNMT5-Flag-R | agtgtcgactctagaggatcctcccatgagtggtcccgtcttttct |
| 2b-1300-F | ccgtcaaacGGATCCCAATTGaggaagttcatttcatttggagagaaca |
| 2b-1300-R | CTGGCGCTTGGTACCGTTAACagttttcccaatgccataatactcgaac |
| pGKO-08181-UP-F | tgaggtcttaattaaGTTAACCCCAGCCAGCGTCTCGTCGAGGG |
| pGKO-08181-UP-R | TTGCGAAGGCGGCTGCAATTGGGTGGTGATGTGTGTGTAAGGGAGGTG |
| pGKO-08181-Dn-F | TGTCTACTGCTGGCCCACGTGTACACTCCGAGACATTGTTGCTGCCTTG |
| pGKO-08181-Dn-R | TAGAACTAGTGGATCCCCGGGTTTCTCAAGTTTTCCAGGCACCGGC |
| pGKO-08177-UP-F | tgaggtcttaattaaGTTAACGCGAAGGGGAGAGGGCATGGGGT |
| pGKO-03177-UP-R | TTGCGAAGGCGGCTGCAATTGGGCGGGGTGGTGATGCTCAGCGG |
| pGKO-03177-Dn-F | TGTCTACTGCTGGCCCACGTGTCCTAATCTTCTCAACTCCTCGTATTGTTC |
| pGKO-03177-Dn-R | TAGAACTAGTGGATCCCCGGGTCAGAACCAAGAATGCCGGTTGTCATC |
| pGKO-RIM15-UP-F | tgaggtcttaattaaGTTAACAAAGGATGGTGGTGGTCGTGGTGGT |
| pGKO-RIM15-UP-R | TTGCGAAGGCGGCTGCAATTGTGTTGAGAGTGACCAGGTTTATTTTGCAGG |
| pGKO-RIM15-Dn-F | TGTCTACTGCTGGCCCACGTGAAATCATGTCGAATGGCGAGCGGG |
| pGKO-RIM15-Dn-R | TAGAACTAGTGGATCCCCGGGAAACGCGTCGACAGAAGACGCCGAA |
| GST-RIM15-F | CCGCGTGGATCCCCGGAATTCACGGCCATAGAAGTAAGCACGCCAG |
| GST-RIM15-R | GTCACGATGCGGCCGCTCGAGTCACAAGGCTTCGGCGCGCAGCTTCT |
| GST-MSN2-F | CCGCGTGGATCCCCGGAATTCgcaccgatgcttcattcgacggata |
| GST-MSN2-R | GTCACGATGCGGCCGCTCGAGtcatgttgctgcgcacgctaaag |
| RIM15 SC-upftank-F | TTTGCGACTCTTTTTAGCCCTACTT |
| RIM15 SC-upftank-R | CTGTCTTCCTCTACTGGGCTTATCT |
| RIM15 SC-Dnftank-F | CGCACTGATAAATTTTAAGATAAAGAT |
| RIM15 SC-Dnftank-R | CTGGGAGAACTATTCTTCCAGAG |
| Leu-F | cgctatcgcacagaatcaaattcg |
| Leu-R | gtatatagtttcgtctaccctatgaaca |
| RIM15 exp in SC-F | cttggtaccgagctcggatccatggacagcaaagatgccagcaaca |
| RIM15 exp in SC-R | tgatggatatctgcagaattctcatttcgaacccggcgtaccgc |
